# Supplementary material for: Greenland ice sheet runoff reduced by meltwater refreezing in bare ice
Source: Nat Commun. 2025 Sep 12;16:8273. doi: 10.1038/s41467-025-62281-0 (PMC12432254; doi:10.1038/s41467-025-62281-0)
Supplement: Supplementary file 1 — Supplementary Information [file 41467_2025_62281_MOESM1_ESM.pdf]

Greenland Ice Sheet runoff reduced by meltwater refreezing in bare ice: Supplementary Information

Matthew G. Cooper[1,2*†], Laurence C. Smith[3,4], Åsa K. Rennermalm[5], Jonathan C. Ryan[6], Lincoln H Pitcher[1], Glen E. Liston[7], Clément Miège[5,8], Sarah W. Cooley[6], Dirk van As[9]

[1]Department of Geography, University of California, Los Angeles, Los Angeles, CA, USA.
[2*]Sierra Crest Analytics, Portland, 97209, OR, USA.
[3]Institute at Brown for Environment and Society, Brown University, Providence, RI, USA.
[4]Department of Earth, Environmental and Planetary Sciences, Brown University, Providence, RI, USA.
[5]Department of Geography, Rutgers, The State University of New Jersey, Piscataway, NJ, USA.
[6]Division of Earth and Climate Sciences, Nicholas School of the Environment, Duke University, Durham, NC, USA.
[7]Cooperative Institute for Research in the Atmosphere, Colorado State University, Fort Collins, CO, USA.
[8]Department of Geography, University of Utah, Salt Lake City, UT, USA.
[9]Geological Survey of Denmark and Greenland, Copenhagen, Denmark.

*Corresponding author(s). E-mail(s): matt@sierracrestanalytics.com;
†The first author performed this work while affiliated with this institution.

Contents

S1 Supplementary Tables **5**

S2 Supplementary Figures **8**

S3 Supplementary Methods **22**

S3.1 IceModel technical description 22

S3.1.1 Heat equation 22

S3.1.2 Two-stream radiative transfer model 25

S3.1.3 Numerical implementation 26

S4 Supplementary Discussion **29**

S4.1 The Role of Albedo Biases in Modulating Runoff Model Errors 29

S4.2 Uncertainty in ice core density measurements 30

S4.3 Uncertainty in Proglacial Discharge Comparisons 31

S4.4 Limitations of the Emulator Approach 32

S5 Supplementary Runoff Comparison Figures **33**

S6 Supplementary Remapping Weight Figures **43**

List of Tables

S1 Catchment surface areas 5

S2 Study datasets 6

S3 Climate model runoff over/underestimation 7

List of Figures

S1 Southwest Greenland study area field sites 8

S2 Field evidence of nocturnal refreezing 9

S3 Catchment area runoff uncertainty for RB catchment in July 2016 . . 10

S4 Surface albedo comparison for RB catchment in July 2015 and 2016 . 11

S5 Surface energy balance for RB catchment in July 2016 12

S6 Surface energy balance for RB catchment in July 2015 13

S7 Climate model runoff comparison for RB catchment in July 2015 . . . 14

S8 Catchment area runoff uncertainty for RB catchment in July 2015 . . 15

S9 Subsurface ice temperatures 16

S10 Surface albedo comparison for the southwest sector from 2009–2018 . 17

S11 Annual average melt, runoff, and refreezing for southwest sector. . . . 18

S12 Conservative regridding of model output onto catchment polgyons . . 19

S13 Scattering coefficients for two-stream radiative transfer model 20

S14 Spectral flux extinction coefficients for glacier ice 21

S15 Runoff comparison: RB catchment in year 2015 33

S16 Runoff comparison: RB catchment in year 2016 34

S17 Runoff comparison: SLV1 catchment in year 2015 34

S18 Runoff comparison: SLV2 catchment in year 2015 35

S19 Runoff comparison: 660 catchment in year 2016 35

S20 Runoff comparison: AK4 catchment over years 2009–2015 36

S21 Runoff comparison: AK4 catchment in year 2009 36

S22 Runoff comparison: AK4 catchment in year 2010 37

S23 Runoff comparison: AK4 catchment in year 2011 37

S24 Runoff comparison: AK4 catchment in year 2012 38

S25 Runoff comparison: AK4 catchment in year 2013 38

S26 Runoff comparison: AK4 catchment in year 2014 39

S27 Runoff comparison: AK4 catchment in year 2015 39

S28 Runoff comparison: LG catchment over years 2009–2012 40

S29 Runoff comparison: LG catchment in year 2009 40

S30 Runoff comparison: LG catchment in year 2010 41

S31 Runoff comparison: LG catchment in year 2011 41

S32 Runoff comparison: LG catchment in year 2012 42

S33 Remapping weights: RB minimum estimated catchment in year 2015 . 43

S34 Remapping weights: RB medium estimated catchment in year 2015 . . 44

S35 Remapping weights: RB maximum estimated catchment in year 2015 . 44

S36 Remapping weights: RB minimum estimated catchment in year 2016 . 45

S37 Remapping weights: RB medium estimated catchment in year 2016 . . 46

S38 Remapping weights: RB maximum estimated catchment in year 2016 . 47

S39 Remapping weights: 660 medium estimated catchment in year 2016 . . 47

S40 Remapping weights: SLV1 minimum estimated catchment in year 2015 48

S41 Remapping weights: SLV1 maximum estimated catchment in year 2015 48

S42 Remapping weights: SLV2 minimum estimated catchment in year 2015 49

S43 Remapping weights: SLV2 maximum estimated catchment in year 2015 49

S44 Remapping weights: AK4 minimum estimated catchment for years 2009–2015 50

S45 Remapping weights: AK4 medium estimated catchment for years 2009–2015 50

S46 Remapping weights: AK4 maximum estimated catchment for years 2009–2015 51

S47 Remapping weights: LG minimum estimated catchment for years 2009–2012 . . . 52
S48 Remapping weights: LG medium estimated catchment for years 2009–2012 . . . 52
S49 Remapping weights: LG maximum estimated catchment for years 2009–2012 . . . 53

S1 Supplementary Tables

Table S1 Catchment surface areas. Catchments with observed discharge or estimated runoff used in this study. Columns indicate catchment names, their lower, upper, and best-estimate surface areas computed in Equal-Area Scalable Earth grid version 2.0 projection (Brodzik et al, 2012), and primary source reference.

	Surface Area [km^2]			
Catchment	lower	upper	best estimate	Reference
RB[1]	50.9	70.8	63.6	(Smith et al, 2017, 2021)
SLV1[1]	10.7	12.6	11.7	(Yang et al, 2019)
SLV2[1]	8.6	8.9	8.8	(Yang et al, 2019)
660[2]	0.53	0.58	0.55	(Muthyala et al, 2022)
AK4[3]	21.8	64.3	33.4	(Rennermalm et al, 2017)
LG[3]	755.6	1223.0	837.1	(Tedstone et al, 2017)

[1]Estimated from digital surface elevation model and satellite image analysis.

[2]Estimated from field mapping of channel divides and satellite image analysis.

[3]Estimated from digital surface and subsurface elevation models and hydraulic potential.

Table S2 Study datasets. Surface-based observations, climate model outputs, and geospatial datasets used in this study.

Dataset type	Time period	Purpose	Reference
RB discharge	20–23 Jul, 2015	Validate modeled runoff	(Smith et al, 2017)
RB discharge	06–13 Jul, 2016	Validate modeled runoff	(Smith et al, 2021)
660 discharge	13 Jun to 13 Aug 2016	Validate modeled runoff	(Muthyala et al, 2022)
AK4 discharge	01 Jun to 31 Aug, 2009–2015	Validate modeled runoff	(Rennermalm et al, 2017)
LG discharge	01 Jun to 31 Aug, 2009–2012	Validate modeled runoff	(Tedstone et al, 2017)
SLV runoff[1]	01 Jun to 31 Aug, 2015	Validate modeled runoff	(Yang et al, 2019)
KAN_M AWS[2]	20–23 Jul, 2015	Model forcing	(Fausto and van As, 2019)
KAN_M AWS	06–13 Jul, 2016	Model forcing	(Fausto and van As, 2019)
KAN_L AWS[3]	01 Jun to 31 Aug, 2009–2016	Model forcing	(Fausto and van As, 2019)
MAR3.11[4]	2009–2018	Model forcing	(Fettweis et al, 2017)
MAR3.11	2009–2018	Modeled runoff	(Fettweis et al, 2017)
MERRA-2	2009–2018	Modeled runoff	(Gelaro et al, 2017)
RACMO2.3p3[5,6]	2012–2018	Modeled runoff	(van Dalum et al, 2021)
RACMO2.3p2[7]	2009–2012	Modeled runoff	(Mankoff et al, 2020)
MODIS[8]	2009–2018	Model albedo forcing	(Box et al, 2017)
IMBIE[9]	–	Southwest sector domain	(Shepherd et al, 2020)
GrSMBMIP[10,12]	–	Bare ice mask	(Fettweis et al, 2020)

[1]Satellite lake volume infilling used as proxy for cumulative runoff.

[2]Used for RB, SLV1, and SLV2 IceModel/Skinmodel simulations depicted in Fig. 2, S7, and S15–S18.

[3]Used for 660 and AK4 IceModel/SkinModel simulations depicted in Fig. S19–S27.

[4]Used for Southwest sector decadal (2009–2018) IceModel/Skinmodel simulations.

[5]RACMO2.3p3 data provided for 2012–2018.

[6]RACMO2.3p3-WIE (Without Internal Energy) data provided for 2012–2015.

[7]RACMO2.3p2 used exclusively for LG runoff comparison (2009–2012) depicted in Fig. S28–S32.

[8]Moderate Resolution Imaging Spectroradiometer MOD10A1 Snow Product.

[9]Ice Sheet Mass Balance Intercomparison.

[10]Greenland ice sheet surface mass balance model intercomparison project.

[11]Bare ice mask from Ryan et al (2019).

Table S3 Climate model runoff over/underestimation. Climate model runoff evaluation, detailed from left to right by the runoff model employed, albedo forcing for the IceModel and SkinModel simulations, runoff over/underestimation for each catchment, expressed as percent differences between cumulative modeled runoff and observed discharge over all years of available data (Table S2), and the average (μ) and standard deviation (σ) across catchments, with rows ordered from high to low average overestimation (μ). The percentage difference (Δ) is assessed between the central estimates of cumulative modeled and observed runoff averaged over the final 24 hours to mitigate the effects of diurnal runoff cycles and routing delays (Methods). Simulations for each site and for each year are depicted in Fig. S15–S32 (Section S5). Missing values correspond to data availability and AWS simulations omitted for LG catchment.

Model	Albedo	Total Cumulative Runoff Over/Underestimation, Δ (%)							
		RB	660	SLV1	SLV2	AK4	LG	μ	σ
SkinModel[1,2]	AWS	42	11	99	111	43	–	**61**	**42**
SkinModel[3]	MODIS	62	12	91	106	31	21	**54**	**39**
RACMO2.3p3-WIE[4]	–	54	–	81	63	38	-2	**47**	**32**
RACMO2.3p2[5]	–	–	–	–	–	53	30	**42**	–
MAR3.11	–	19	21	77	63	45	8	**39**	**27**
RACMO2.3p3[6]	–	58	5	76	58	37	-5	**38**	**32**
SkinModel	MAR3.11	3	9	91	81	29	-7	**34**	**42**
MERRA-2	–	-7	-37	69	65	-20	-17	**9**	**46**
IceModel[1]	MODIS	31	-14	-13	7	-6	-15	**-2**	**18**
IceModel[2,3]	AWS	2	-37	-4	1	-6	–	**-9**	**16**
IceModel	MAR3.11	-18	-16	-23	-42	-7	-39	**-24**	**14**

[1]KAN_M AWS albedo used for RB, SLV1, and SLV2 forcings.

[2]KAN_L AWS albedo used for 660 and AK4 forcings.

[3]Moderate Resolution Imaging Spectroradiometer MOD10A1 land ice albedo (Box et al, 2017).

[4]RACMO2.3p3-WIE evaluated for year 2012 at LG and 2012–2015 at AK4.

[5]RACMO2.3p2 evaluated for years 2009–2012 at LG and 2009–2015 at AK4.

[5]RACMO2.3p3 evaluated for years 2012–2018.

S2 Supplementary Figures

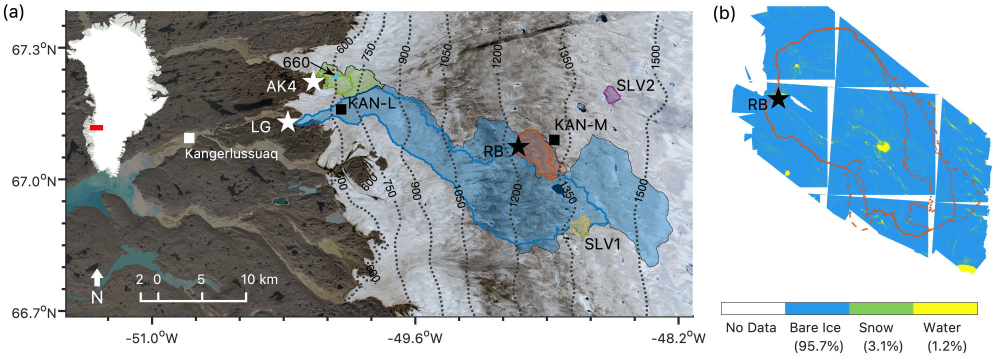

Fig. S1 Southwest Greenland study area field sites. (a) Study area map showing the RB catchment boundary and discharge gauge site location (black star) ($67.05° N$, $-49.02° W$; 1215 m a.s.l.), automatic weather stations KAN_M ($67.067° N$, $-48.835° W$; 1270 m a.s.l.) and KAN_L ($67.096° N$, $-49.951° W$; 672 m a.s.l.), Leverett Glacier (LG) catchment and discharge gauge site location (white star), Akuliarusiarsuup Kuua River's northern tributary (AK4) catchment and discharge gauge site location (white star), supraglacial catchment 660 nested within AK4, and two supraglacial lake catchments with satellite lake volume (SLV) estimates (Yang et al, 2019). Filled catchment polygons represent the upper estimate of the catchment boundary contributing area; lower estimates are depicted as solid lines nested within filled polygons. Background image is Landsat 8 true color composite on 26 July 2016. (b) Surface classification for the RB catchment from UAV survey imagery during the 6–13 July 2016 field experiment. Also displayed are lower (dotted line), upper (dashed line), and "best guess" (solid line) catchment boundaries, with areas of 50.9, 70.8, and 63.6 km^2, respectively (Table S1).

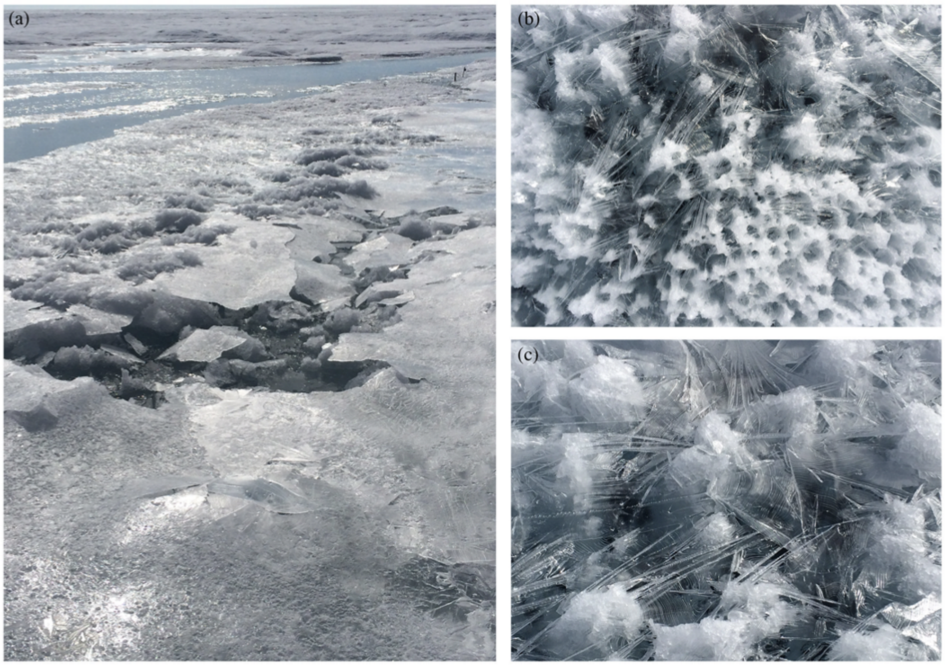

Fig. S2 Field evidence of nocturnal refreezing. Night-time refreezing of meltwater was frequently observed during the July 2016 field study, shown here at the surface of (a) water tracks (∼10 m length scale), and (b) within weathering crust cryoconite holes (∼0.1–1 m length scale). Photos were collected by the first author during the 6–13 July 2016 field study between 04:00 and 07:00 local time (UTC-2). Reprinted with permission from Cooper et al (2018) (doi.org/10.5194/tc-12-955-2018, CC BY 4.0). See also Fig. 3 (Main).

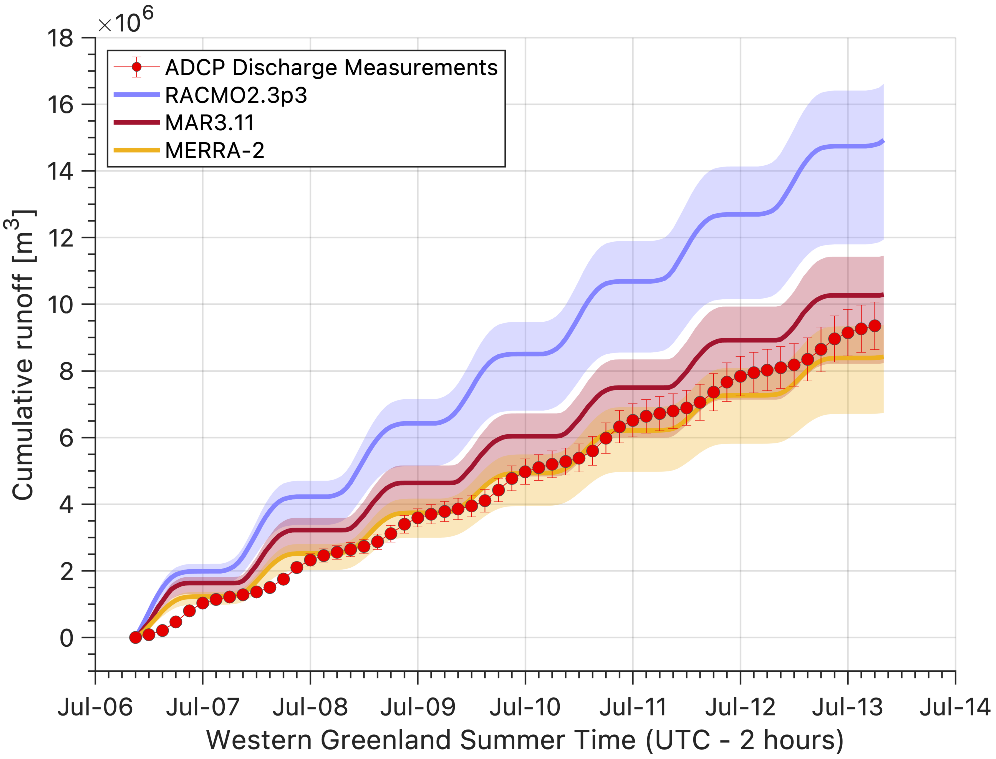

Fig. S3 Catchment area runoff uncertainty for RB catchment in July 2016. Similar to Fig. 2 (Main), this figure presents catchment area uncertainty for climate model meltwater runoff predictions for the July 2016 field study. Area uncertainty for SkinModel and IceModel is depicted in Fig. 2, and excluded here for visual clarity.

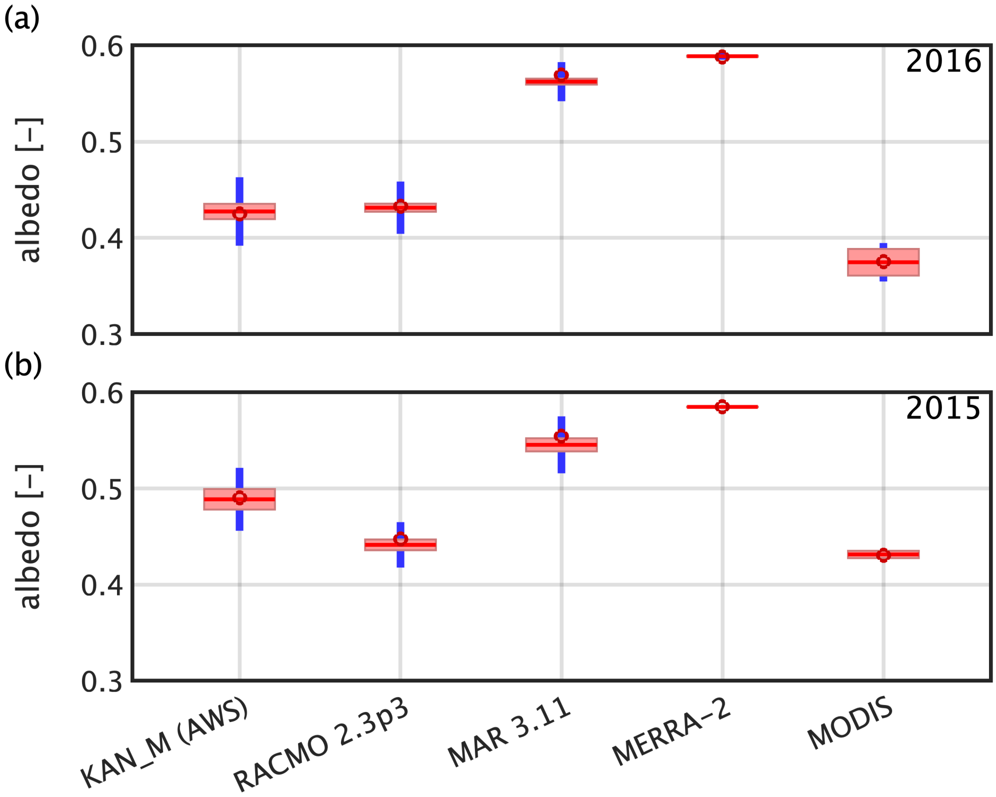

Fig. S4 Surface albedo comparison for RB catchment in July 2015 and July 2016. Comparison of observed hourly albedo from the KAN_M automatic weather station (AWS) with outputs from two regional climate models (RACMO2.3p3 and MAR3.11), a global climate reanalysis (MERRA-2), and daily albedo from the MODIS satellite. This comparison covers two periods: (a) the 6–13 July 2016 and (b) the 20–23 July 2015 field experiments. RACMO2.3p3 aligns most closely with observed albedo values, consistent with observed net radiation (Fig. S5–S6), but tends to overestimate meltwater runoff (Fig. S8 and Fig. 2, Main). In contrast, MAR3.11 and MERRA-2 overestimate albedo, explaining the apparent agreement between their simulated runoff and observed discharge (Fig. S8 and Fig. 2, Main). A similar comparison for the southwest sector is shown in Fig. S10. Each box chart shows the mean (solid line), median (open circle), standard error of the mean (distance between the upper and lower box edges), and two standard deviations of the data (distance from the bottom of the lower whisker to the top of the upper whisker)

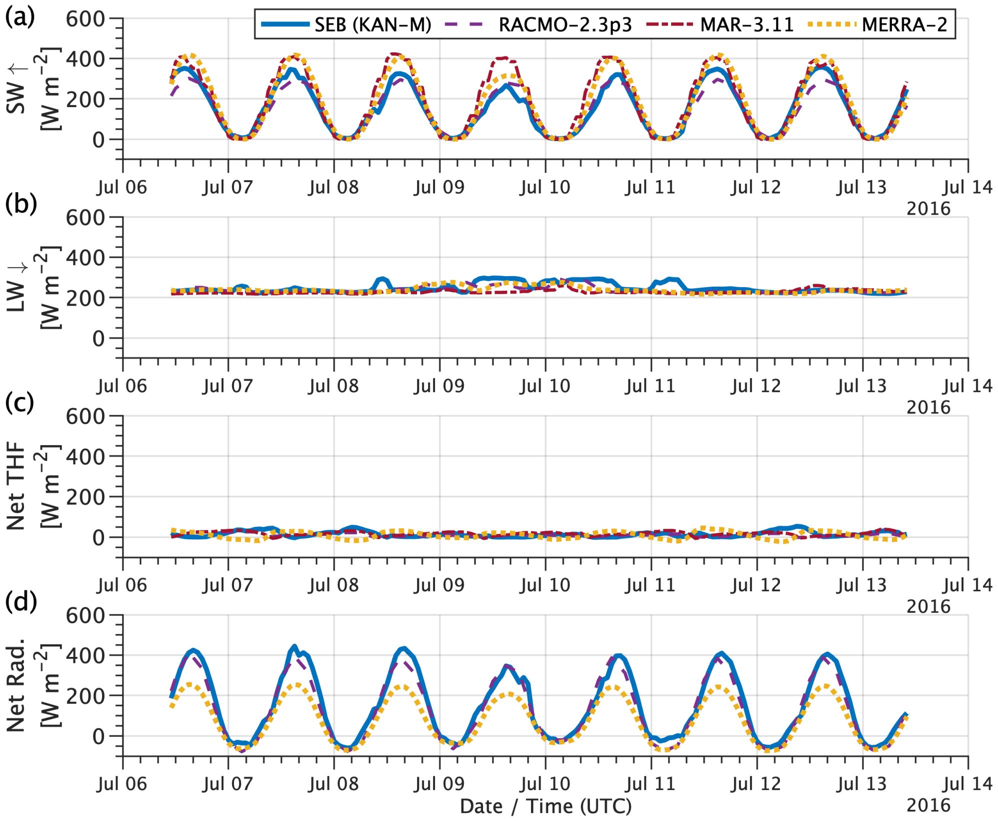

Fig. S5 Surface energy balance during July 2016 in RB catchment. Hourly values of (a) reflected shortwave radiation flux, (b) downward longwave radiation flux, (c) net turbulent heat flux, and (d) net radiation, averaged over grid cells intersecting the RB catchment, from 6 to 13 July 2016. Data sources include MERRA-2, RACMO2.3p3, MAR3.11, and an independent offline surface energy balance (SEB) model (van As, 2011) forced by KAN_M weather station observations.

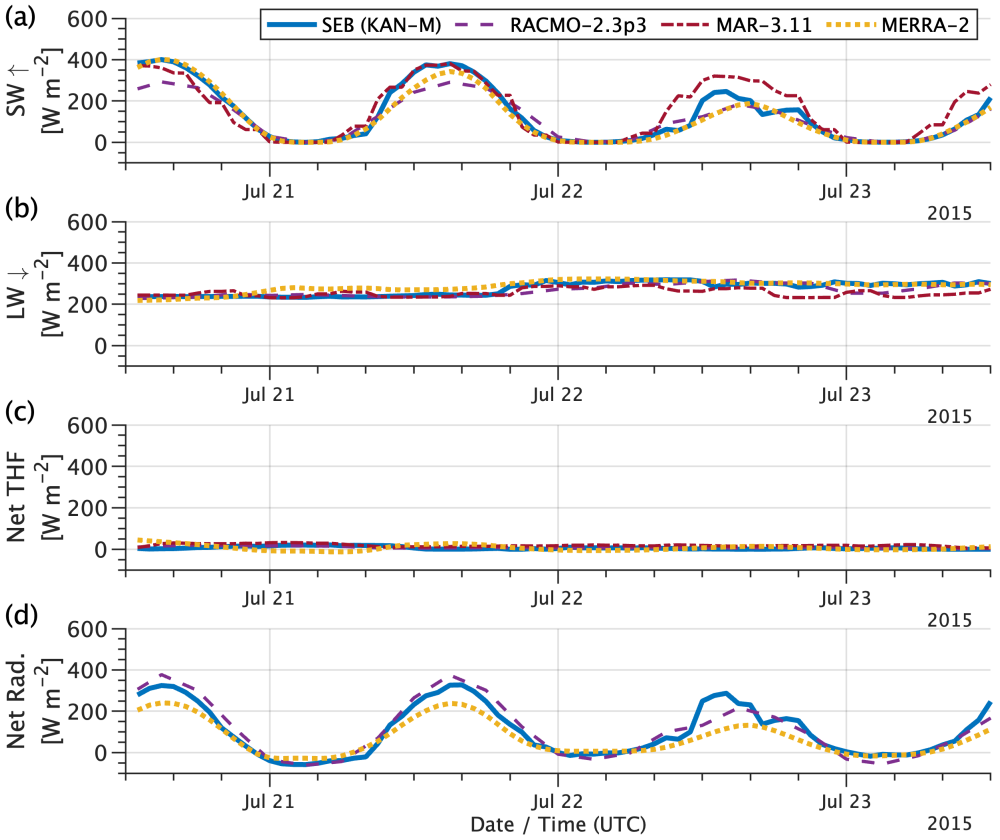

Fig. S6 Surface energy balance for RB catchment in July 2015. Hourly values of (a) reflected shortwave radiation flux, (b) incoming longwave radiation flux, (c) net turbulent heat flux, and (d) net radiation, averaged over grid cells intersecting the RB catchment, from 20 to 23 July 2015. Data sources include MERRA-2, RACMO2.3p3, MAR3.11, and an independent offline surface energy balance (SEB) model (van As, 2011) forced by KAN_M weather station observations.

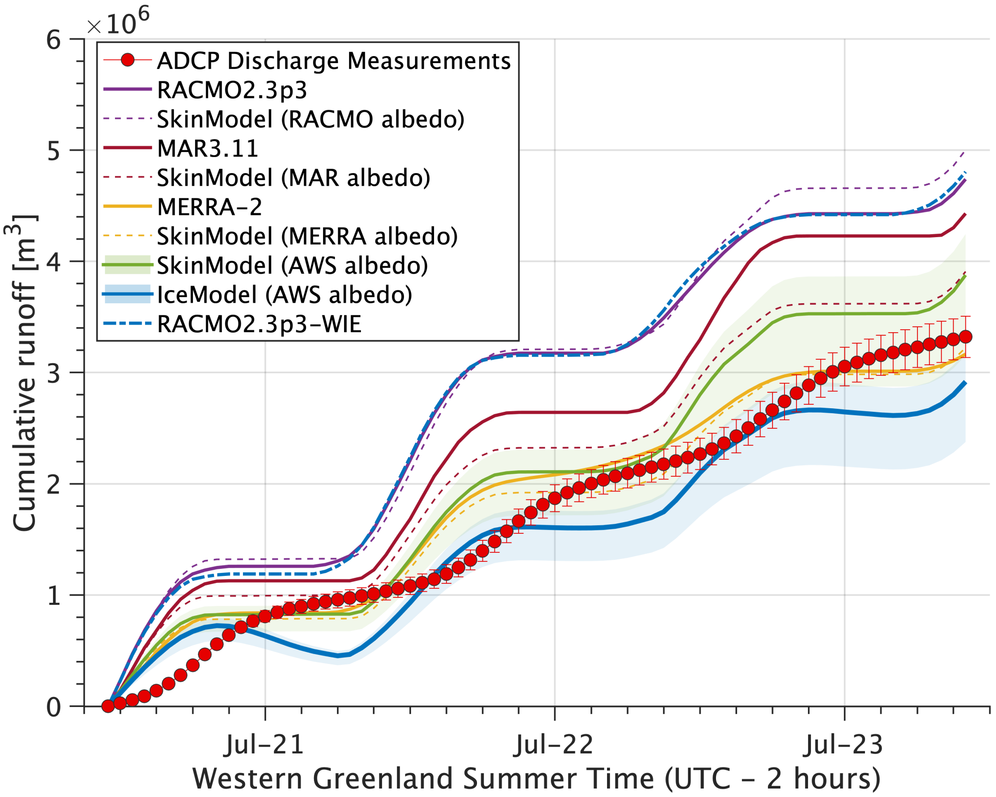

Fig. S7 Climate model runoff compared to observed runoff in RB catchment in July 2015. Cumulative values of meltwater runoff measured with an acoustic Doppler current profiler (ADCP) compared to runoff predictions from three climate models and simulations from SkinModel and IceModel during the 20–23 July 2015 field experiment (Smith et al, 2017). Dashed lines depict runoff predictions from SkinModel, our climate model surface energy balance emulator, deliberately forced with albedo outputs from each climate model. Solid lines with shaded bounds represent runoff predictions from SkinModel and IceModel, both driven by actual albedo recorded by the KAN_M automatic weather station (AWS). Solid and dashed lines indicate central runoff estimates for the 63.6 km^2 catchment area, with shaded bounds reflecting area uncertainty (50.9 to 70.8 km^2) (the catchment area uncertainty for each climate model is shown in Fig. S8). Error bars represent two standard deviations of the ADCP discharge. Major ticks and dates are at 00:00 local time (UTC-2).

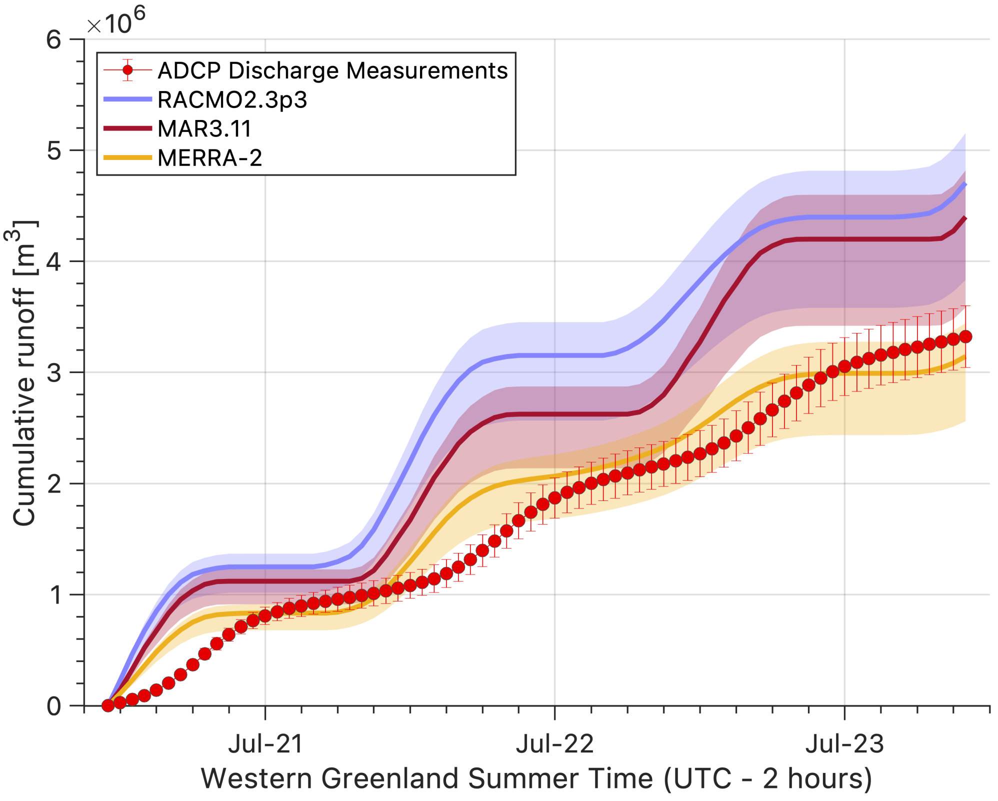

Fig. S8 Catchment area runoff uncertainty for RB catchment in July 2015. Similar to Fig. S7, this figure presents catchment area uncertainty for climate model meltwater runoff predictions for the July 2016 field study. Area uncertainty for SkinModel and IceModel is depicted in Fig. S7, and excluded here for visual clarity.

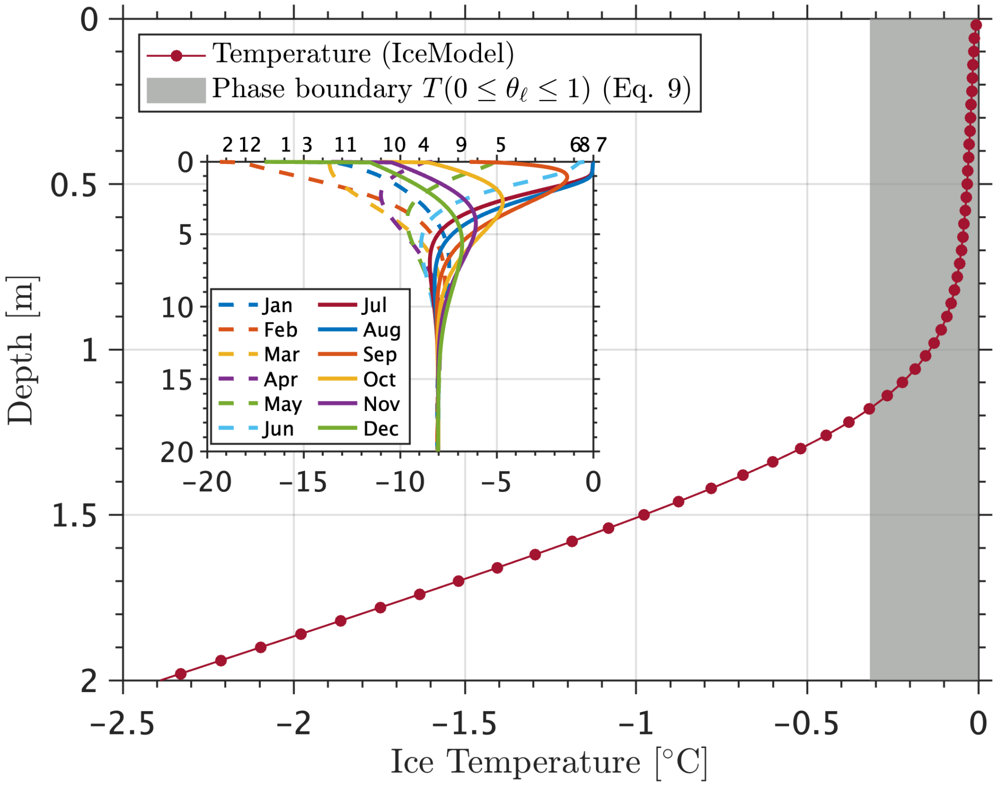

Fig. S9 Subsurface ice temperatures. Subsurface ice temperatures in the top 2 m during the 6–13 July 2016 field campaign, simulated using IceModel with KAN_M weather station forcings. The gray shaded area indicates the temperature range over which phase change occurs, with the left and right edges representing the temperatures at which the liquid water content is approximately zero and one, respectively. Temperatures represent average values between 10:00 and 18:00 local time, to demonstrate subsurface radiative heating due to transmitted solar radiation during daylight hours. The inset displays monthly average temperature profiles in the top 20 m of ice, with month numbers (1–12: Jan–Dec) shown along the top edge. Jan–Jun curves are dashed lines, and Jul–Dec curves are solid lines.

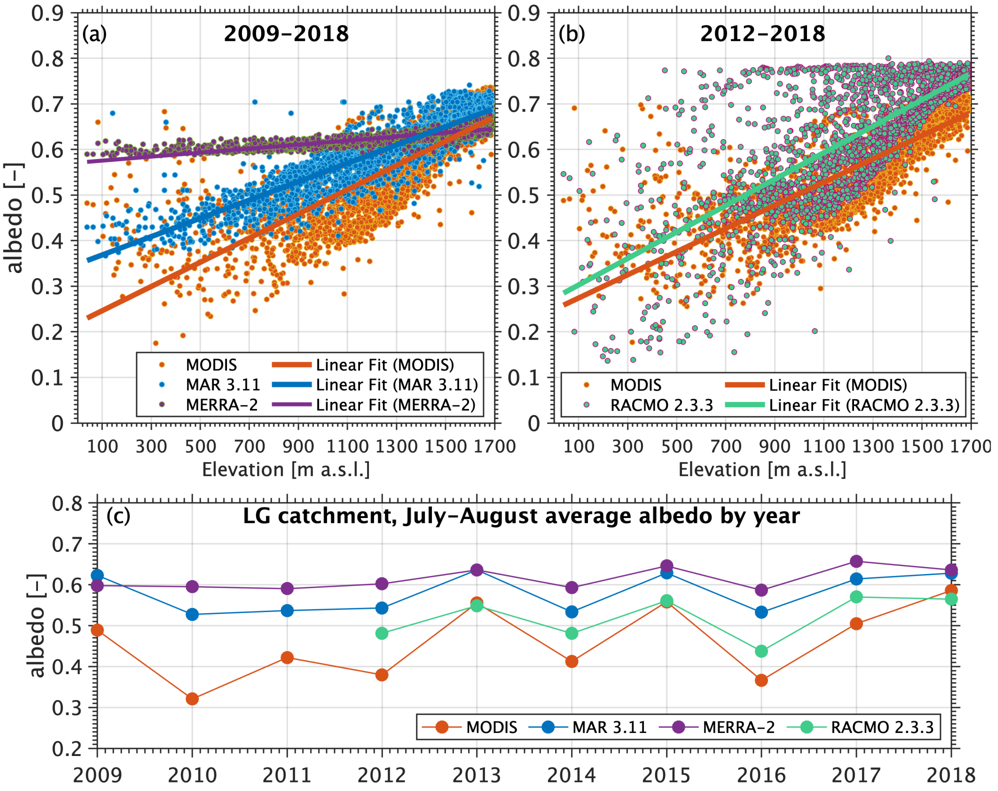

Fig. S10 Surface albedo comparison for Greenland's southwest sector from 2009–2018. Panel (a) presents mean daily albedo values during July and August, 2009–2018, from MODIS observations alongside values from MAR3.11 and MERRA-2. Panel (b) presents a similar comparison between MODIS albedo and RACMO2.3p3 albedo for 2012–2018, corresponding to the availability of RACMO2.3p3 data. MAR3.11 and MERRA-2 are omitted from panel (b) for clarity, although their trends are qualitatively similar to those in panel (a). Data points in panels (a) and (b) represent 5-km grid cell average albedos within the Ice Sheet Mass Balance Intercomparison Exercise (IMBIE) (Shepherd et al, 2020) Southwest Sector, restricted to grid cells classified as bare ice in the Greenland Ice Sheet Surface Mass Balance Model Intercomparison Project (GrSMBMIP) (Fettweis et al, 2020). Solid lines depict the least-squares fit correlating surface albedo with elevation (in meters above sea level, m a.s.l.). Panel (c) shows the July–August average albedo for each year from 2009–2018 for LG catchment, the largest in this study. For interpretation of RACMO's steeper albedo trend relative to MODIS in panel (b), see Section S4.1.

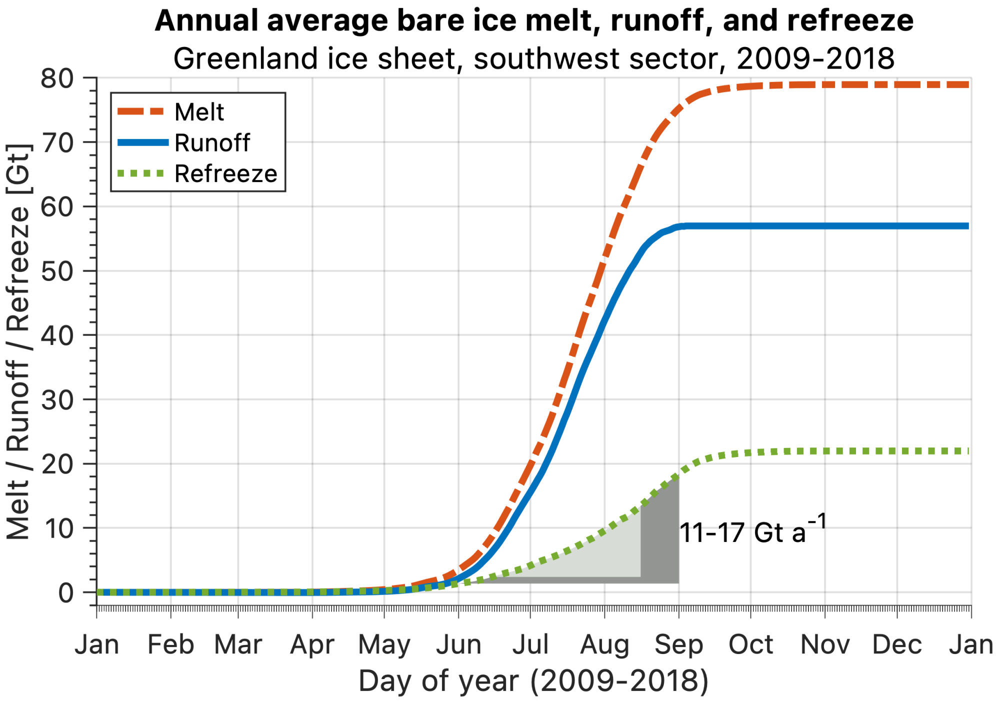

Fig. S11 Annual average melt, runoff, and refreezing for southwest sector. Annual average meltwater production, refreezing, and runoff from IceModel simulations forced with MODIS surface albedo for the southwest sector bare-ice domain. The gray shaded areas depict two estimates of the annual average runoff reduction due to refreezing in bare ice: a "lower" (15 June to 15 August) proxy for seasonal time of bare-ice exposure, and an "upper" one (1 June to 31 August). IceModel runoff and refreezing estimates for these two time periods were calculated directly from the regional-scale gridded simulations, restricted to grid cells within the MODIS-derived bare-ice mask (Methods).

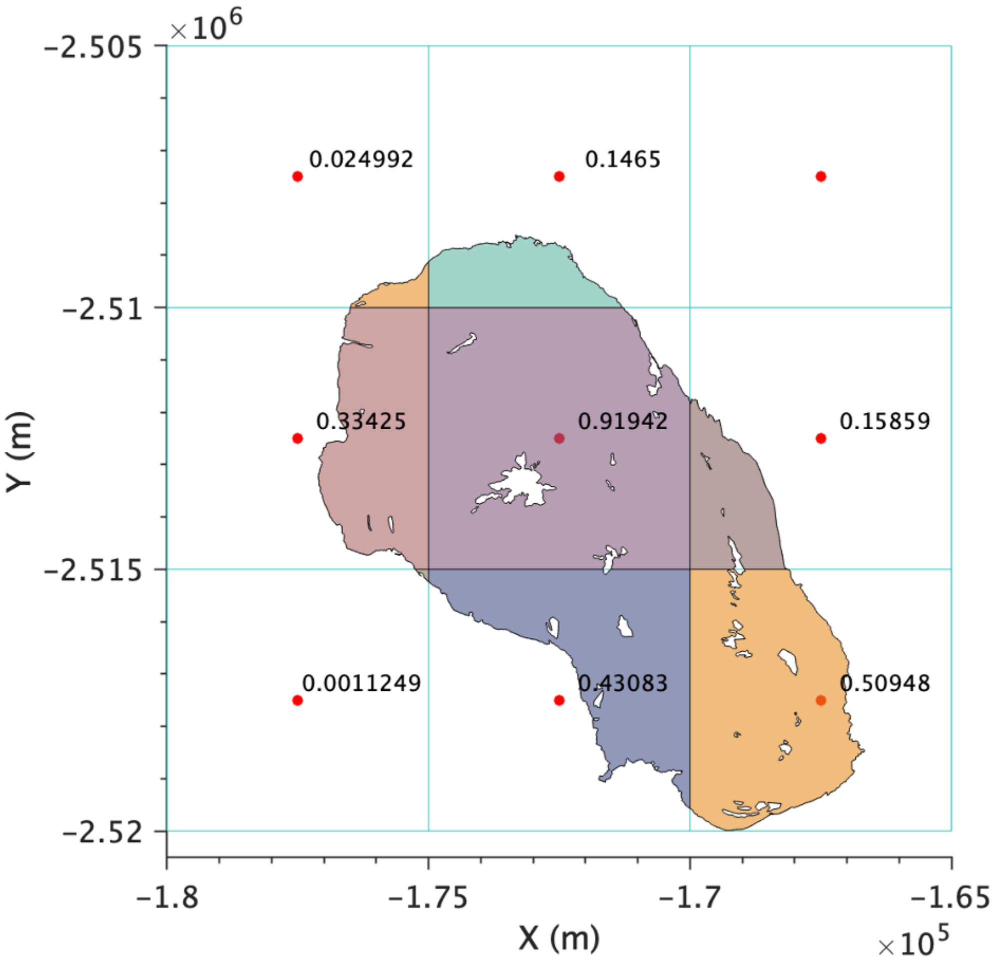

Fig. S12 Conservative remapping of model output onto catchment polgyons. Example of conservative remapping used to compute catchment area-weighted values of gridded model output. This figure depicts a regular grid with 5 km horizontal resolution projected onto the WGS 84 / NSIDC Sea Ice Polar Stereographic North coordinate system, intersected with the RB catchment boundary to compute remapping weights for conversion of specific meltwater runoff to catchment area-weighted volumetric runoff. Grid cell centroids (red circles) are labeled with associated remapping weights, which indicate the overlapping area of each grid cell and the catchment polygon. The catchment boundary shown here contains areas drained by crevasse fields and internal moulins (depicted as interior holes) which are subtracted from the contributing area of the catchment (Methods). Similar figures for each catchment analyzed in this study are included in Sec. S6.

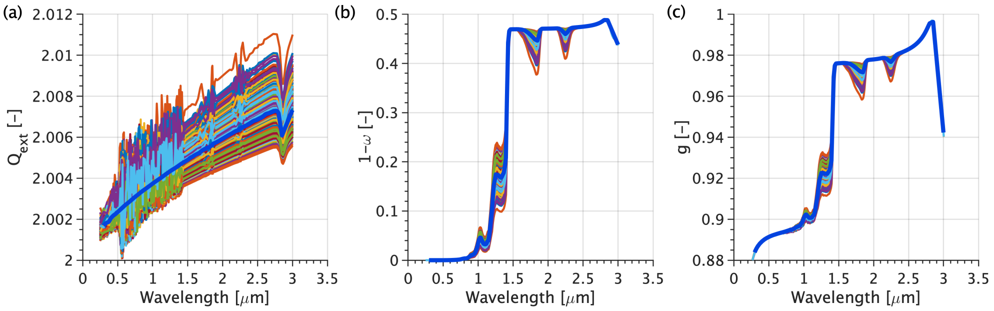

Fig. S13 Scattering coefficients for two-stream radiative transfer model. (a) Single-scattering extinction coefficient Q_{ext}, (b) single-scattering co-albedo $1 - \omega$, and (c) asymmetry parameter g, computed using Mie scattering algorithms for a grain size ensemble ($N = 1000$) following a normal distribution with a mean of 2.0 mm and a standard deviation of 0.3 mm. Ensemble averages for 118 spectral bands covering the solar spectrum (0.3–3.03 μm) are displayed as thick blue lines.

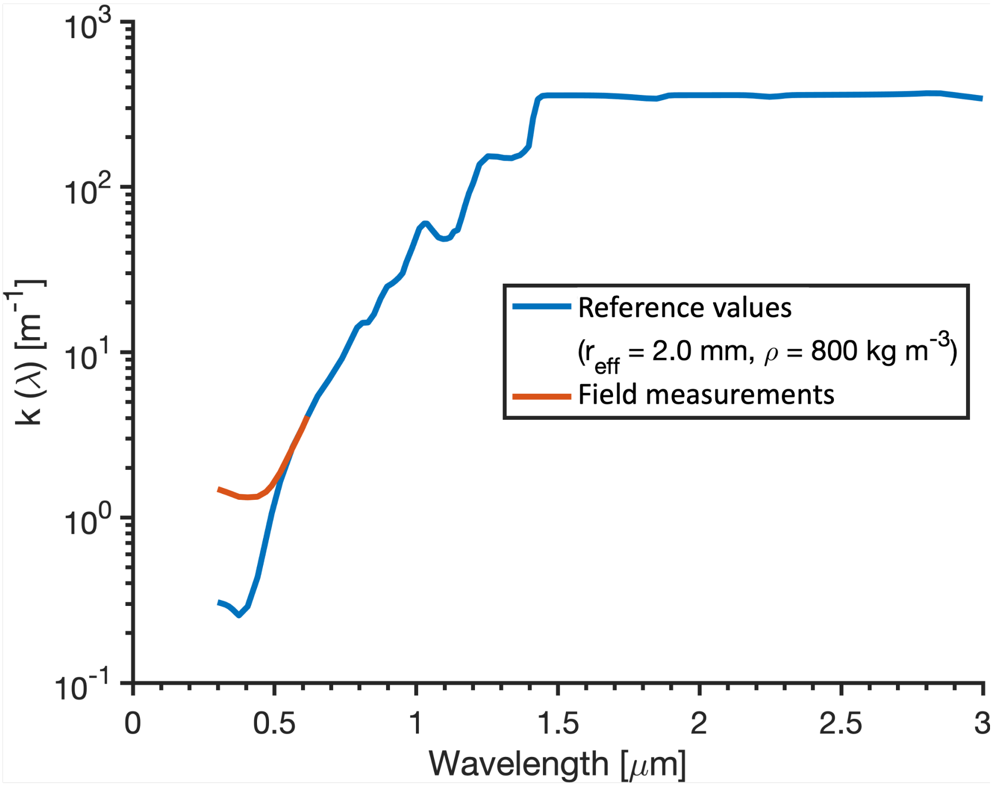

Fig. S14 Spectral flux extinction coefficients for glacier ice. Values of the flux extinction coefficient $k(\lambda)$ from 0.3–3.0 μm, for an ice volume with an effective optical grain radius of r_{eff} = 2.0±0.3 mm and a bulk density of $\rho = 800\,\text{kg/m}^3$. Reference values for these coefficients are calculated using Equation 20 and the single scattering coefficients presented in Fig. S13 for an optically pure glacier ice volume. The observed values are from Equation 22 using ice absorption coefficient values derived from measurements in the study area (Cooper et al, 2021), with elevated values in the spectral region 0.3–0.7 μm attributed to the presence of light-absorbing impurities within the ice.

S3 Supplementary Methods

S3.1 IceModel technical description

S3.1.1 Heat equation

IceModel solves the unsteady one-dimensional heat equation:

$$\frac{\partial H}{\partial t} = \frac{\partial F}{\partial z} + S \tag{1}$$

where H [J m^{-3}] is enthalpy per unit volume, t [s] is time, z [m] is the vertical dimension (positive down), F [W m^{-2}] is net heat flux along the vertical dimension, and S [W m^{-3}] is a heat source/sink term. The enthalpy of the mixture of ice, liquid water and water vapor is defined as (Jordan, 1991):

$$H = \int_{T_{\mathrm{ref}}}^{T} \left(\rho_i \theta_i c_i + \rho_\ell \theta_\ell c_\ell + L_f \rho_\ell \frac{\partial \theta_\ell}{\partial T} + L_v \theta_v \frac{\partial \rho_v}{\partial T} \right) dT. \tag{2}$$

Here, T [K] is temperature, $T_{\mathrm{ref}} = 273.16$ K is the triple-point temperature of water, ρ_i [kg m^{-3}] is ice density, ρ_ℓ [kg m^{-3}] is liquid water density, θ_i [m^3 m^{-3}] is volumetric ice fraction, θ_ℓ [m^3 m^{-3}] is volumetric liquid water fraction, c_i [J kg^{-1} K^{-1}] is specific heat capacity of ice, c_ℓ [J kg^{-1} K^{-1}] is specific heat capacity of liquid water, L_f [J kg^{-1}] is latent heat of fusion of ice, $\rho_{v,\mathrm{sat}}$ [kg m^{-3}] is saturation water vapor density, L_v [J kg^{-1}] is latent heat of vaporization of ice, and θ_v [m^3 m^{-3}] is volumetric water vapor fraction. Equation 2 neglects sensible heat effects associated with water vapor and dry air, and assumes that the system is isolated from work interactions with its surroundings.

Fluxes across internal layers ($z > z_{\mathrm{sfc}}$) are driven by heat conduction and vapor diffusion through the ice matrix:

$$F(z > z_{\mathrm{sfc}}) = -k_e \frac{\partial T}{\partial z} \tag{3}$$

where the mixture effective thermal conductivity, k_e [W m^{-1} K^{-1}], is:

$$k_e = \theta_i k_i + \theta_\ell k_\ell + \theta_v k_v. \tag{4}$$

Here, k_ℓ [W m^{-1} K^{-1}] is the thermal conductivity of liquid water, the thermal conductivity of the ice, k_i, is modeled by Equation 5 of Calonne et al (2019), and the thermal diffusivity of the vapor, k_v [W m^{-1} K^{-1}], is

$$k_v = D_e L_v \frac{\partial \rho_{v,\mathrm{sat}}}{\partial T} \tag{5}$$

where,

$$\frac{\partial \rho_{v,\mathrm{sat}}}{\partial T} = \frac{1}{R_v T}\left(\frac{\partial P_{v,\mathrm{sat}}}{\partial T} - \frac{P_{v,\mathrm{sat}}}{T}\right). \tag{6}$$

The vapor diffusivity, D_e [m^2 s^{-1}], is given by Anderson (1976):

$$D_e = 9.0 \times 10^{-5} \left(\frac{T}{T_{\mathrm{ref}}}\right)^{n_D}, \tag{7}$$

and the saturation vapor pressure, $P_{v,\mathrm{sat}}$ [Pa], is defined following Buck (1981):

$$P_{v,\mathrm{sat}} = P_{v0,\mathrm{sat}} \exp\left[\frac{b(T - T_{\mathrm{ref}})}{c + T - T_{\mathrm{ref}}}\right]. \tag{8}$$

The liquid water fraction, θ_ℓ, is related to the depression temperature, $T_D = T_{\mathrm{ref}} - T$, by the phase fraction characteristic function (Jordan, 1991; Clark et al, 2021):

$$\frac{\theta_\ell}{\theta_w} = \frac{1}{1 + [a(T_{\mathrm{ref}} - T)]^2}, \tag{9}$$

and,

$$\frac{\partial \theta_l}{\partial T} = 2a^2 (T_{\mathrm{ref}} - T)(\theta_\ell^2/\theta_w) \tag{10}$$

where,

$$\theta_w = \theta_\ell + \theta_i(\rho_i/\rho_\ell) \tag{11}$$

is the volumetric total water fraction.

The heat flux at the surface, $F(z = z_{\mathrm{sfc}})$ [W m^{-2}], couples the subsurface ice column to the atmosphere:

$$F(z = z_{\mathrm{sfc}}) = Q_\downarrow^{SW}(1 - \alpha)\chi + \varepsilon(Q_\downarrow^{LW} - \sigma T_{\mathrm{sfc}}^4) + Q_H + Q_E + Q_c \tag{12}$$

where,

$$Q_c = k_e \frac{\partial T}{\partial z}\bigg|_{z = z_{\mathrm{sfc}}} \tag{13}$$

is the conductive heat flux, $Q_\downarrow^{SW}$ is the downward shortwave (solar) radiation flux, α [-] is the surface albedo, χ [-] is the fraction of the net solar radiation flux absorbed in the surface layer, ε [-] is the surface emissivity, $Q_\downarrow^{LW}$ is the downward longwave radiation flux, σ [W m^{-2} K^{-4}] is the Stefan-Boltzmann constant, and T_{sfc} [K] is the surface temperature. The sensible heat flux Q_H, and the latent heat flux Q_E, are calculated using Monin-Obukhov similarity theory following methods described in Liston et al (1999). All fluxes in Equation 12 are defined as positive into the surface.

S3.1.2 Two-stream radiative transfer model

Subsurface absorption of transmitted solar radiation is represented by the source term:

$$S(z > z_{\rm sfc}) = -\frac{\partial Q_s}{\partial z} \tag{14}$$

where the subsurface net radiative flux, Q_s [W m^{-2}], is modeled with the two-stream radiative transfer model described by Schlatter (1972), updated with spectral detail following Brandt and Warren (1993):

$$Q_s(z) = Q^{\downarrow} - Q^{\uparrow} \tag{15}$$

$$\mathrm{d}Q^{\uparrow} = (aQ^{\uparrow} + rQ^{\uparrow} - rQ^{\downarrow})\mathrm{d}z \tag{16}$$

$$\mathrm{d}Q^{\downarrow} = (-aQ^{\downarrow} - rQ^{\downarrow} + rQ^{\uparrow})\mathrm{d}z \tag{17}$$

$$a = \eta(z)\left(\frac{1-\alpha}{1+\alpha}\right), \quad r = \eta(z)\left(\frac{2\alpha}{1-\alpha^2}\right), \tag{18}$$

where $Q^{\uparrow}$ and $Q^{\downarrow}$ are the upward and downward fluxes at level z, respectively, and a and r are the absorptivity and reflectivity of level z, respectively. The spectrally integrated downward flux extinction coefficient, $\eta(z)$ [m^{-1}], is given by Brandt and Warren (1993):

$$\eta(z) = -\frac{1}{\Delta z}\ln\left[\frac{\int Q_\lambda^{\downarrow}\exp[-k_\lambda(z+\Delta z)]\mathrm{d}\lambda}{\int Q_\lambda^{\downarrow}\exp[-k_\lambda(z)]\mathrm{d}\lambda}\right] \tag{19}$$

where $Q_\lambda^{\downarrow}$ [W m^{-2} nm^{-1}] is the downward spectral shortwave radiation flux at the ice surface for wavelength λ, k_λ [m^{-1}] is the spectral flux extinction coefficient for wavelength λ:

$$k(\lambda) = \sigma_{\rm ext}(\lambda)\sqrt{(1-\omega(\lambda))(1-g(\lambda)\omega(\lambda))}, \tag{20}$$

and,

$$\sigma_{\text{ext}}(\lambda) = \frac{3}{4}\frac{Q_{\text{ext}}(\lambda)}{r_{\text{eff}}} \tag{21}$$

is the single-scattering extinction coefficient. Values for the single-scattering extinction efficiency $Q_{\text{ext}}(\lambda)$, the co-albedo $1 - \omega(\lambda)$, and the scattering anisotropy $g(\lambda)$ were calculated as functions of grain size, r_{eff}, using Mie scattering algorithms provided as MATLAB code by Mätzler (2002) and the complex index of refraction of pure ice from Warren and Brandt (2008). The Mie solutions at each wavelength were integrated over a Gaussian size distribution ($N = 1000$) of scattering radii $\mathcal{N}(\mu = 2.0\,\text{mm}, \sigma = 0.3\,\text{mm})$ to eliminate ripples associated with the Bessel function solutions to the Mie equations (Fig. S13) (Bohren and Huffman, 2007).

Equation 20 represents the spectral flux extinction in a scattering and absorbing medium composed of ice and liquid water. Absorption of visible light by impurities contained within the ice matrix is represented by Equation 15 of Warren et al (2006):

$$k(\lambda) = k(\lambda_0)\sqrt{\frac{k_{\text{abs}}(\lambda)}{k_{\text{abs}}(\lambda_0)}}, \quad 300 < \lambda < 700\,\text{nm} \tag{22}$$

where $\lambda_0 = 600\,\text{nm}$ is a reference wavelength. Values for $k_{\text{abs}}(\lambda)$ used as input to Equation 22 were obtained from measurements of spectral flux extinction within glacier ice in Greenland's western ablation zone (Fig. S14) (Cooper et al, 2021).

S3.1.3 Numerical implementation

Following Patankar (1980), we develop a conservative finite volume discretization of the heat equation, by integrating Equation 1 over a control volume of thickness Δz, and a time interval from t to $t + \Delta t$:

$$\int_{z}^{z+\Delta z}\int_{t}^{t+\Delta t}\frac{\partial H}{\partial t}\,dt\,dz = \int_{t}^{t+\Delta t}\int_{z}^{z+\Delta z}\frac{\partial F}{\partial z}\,dz\,dt + S, \tag{23}$$

which yields the following discretized equation under a fully-implicit time integration:

$$\Delta H \Delta z = \left(k_e \frac{\mathrm{d}T}{\mathrm{d}z} \right) \Delta t + \Delta Q_s \Delta t. \tag{24}$$

The change in enthalpy, ΔH [J m^{-3}], is represented by a Taylor expansion (Swaminathan and Voller, 1993; Clark et al, 2021):

$$\Delta H^{i+1} = \Delta H^{i} + \left(\frac{\mathrm{d}H}{\mathrm{d}T} \right)^{i} \delta T^{i+1} \tag{25}$$

where i indexes nonlinear iterations within a timestep, $\delta T^{i+1} = T^{i+1} - T^{i}$ is the temperature change over one iteration, and $\Delta H^{i} = H^{i} - H^{o}$ is the enthalpy change relative to the start of a timestep, where o indexes "old" values, prior to the first iteration.

Substituting the expanded enthalpy expression into Equation 24, we obtain the following conservative discretization of the heat equation:

$$\Delta H^{i} + \left(\frac{\mathrm{d}H}{\mathrm{d}T} \right)^{i} \delta T^{i+1} = \frac{\Delta t}{\Delta z} \left(k_e \frac{\mathrm{d}T}{\mathrm{d}z} + \Delta Q_s \right)^{i} \tag{26}$$

where $\mathrm{d}H/\mathrm{d}T$ is given by the integrand of Equation 2.

Equation 26 is equivalent to the optimal enthalpy scheme defined by Equations 17–18 and Equation 24 of Swaminathan and Voller (1993). Operationally, Equation 26 is cast in a tridiagonal form and solved for the current temperature field, T^{i+1}, which is then corrected to be consistent with the current enthalpy field, H^{i+1}, following Equation 25 (Swaminathan and Voller, 1993).

Following each successful iteration, if the ice fraction θ_i of the upper layer drops below a prescribed 10% threshold, it merges with the layer directly beneath it. Like

Equation 26, this combination process is governed by the principle of enthalpy conservation. Following Equation 139 of Jordan (1991), the sum of the enthalpies of the individual layers is equated to the enthalpy of the combined layer. This calculation results in a third-order polynomial equation in terms of the temperature of the combined layer. The root of the polynomial is efficiently located using a derivative-free solver based on Brent's method.

The system of equations is solved on a uniform mesh with a 4 cm node spacing extending from a surface datum ($z = z_{\mathrm{sfc}}$) to a depth of 20 m. The spectral model provides the source term ΔQ_s on a uniform mesh with a 2 mm node spacing extending from the surface to a depth of 12 m, following the method of solution detailed in Appendix 1 of Schlatter (1972). At the upper boundary, Equation 12 is solved for the ice surface temperature T_{sfc} using Newton-Rhapson iteration. At the lower boundary, a zero-flux condition is assumed:

$$k_e \frac{\partial T}{\partial z}\bigg|_{z=z_{\max}} = 0 \tag{27}$$

where an initial value $T(z_{\max}) = -8.0$ ◦C is prescribed from measurements of ice temperature in Greenland's western ablation zone (Hills et al, 2018).

S4 Supplementary Discussion

S4.1 The Role of Albedo Biases in Modulating Runoff Model Errors

The relationship between modeled runoff accuracy and model physics becomes clearer when considering how albedo biases influence melt production. At all sites, IceModel forced with MODIS albedo predicts systematically lower runoff than SkinModel forced with MODIS (Table S3), consistent with the expected runoff reduction due to bare ice refreezing. However, IceModel forced with MAR albedo underpredicts runoff, while MAR itself generally overpredicts or appears to match observations due to a compensating albedo bias that suppresses melt energy—an effect that, in reality, may have been caused by refreezing. This highlights that models can arrive at the right answer for the wrong reasons, a distinction often overlooked in evaluations of climate model performance.

Elevation further modulates these effects. The strongest climate model runoff overestimations occur at high-elevation SLV sites, where refreezing is expected to be most significant, while biases are smaller at lower elevations, such as AK4 and 660. LG catchment integrates runoff from both low and high elevations, complicating direct attribution of runoff biases to albedo alone, but follows the same overall pattern: higher runoff when MODIS albedo is used and lower runoff when MAR albedo is used; higher runoff when SkinModel is used and lower runoff when IceModel is used.

The importance of albedo in driving model runoff biases is further evident in MERRA-2, which exhibits a strong positive albedo bias at low elevations that diminishes and then reverses at high elevations (Fig. S10). This pattern is directly reflected in MERRA-2's runoff biases, which are strongly negative at low-elevation sites and strongly positive at high-elevation sites SLV1 and SLV2 (Table S3). All of this reinforces the conclusion that bare ice refreezing reduces effective meltwater export, an

effect that is underestimated in climate models and often masked by compensating albedo biases.

Finally, it is important to interpret the albedo-elevation relationships fro the SW sector shown in Fig. S10 carefully, as the grid cells used in this analysis are restricted to bare ice areas identified by our MODIS-derived bare ice mask. RACMO's trend line is notably steeper at elevations above ∼1100 m, which might lead one to conclude that RACMO's bare-ice albedo systematically exceeds that of MAR and/or MERRA-2. However, RACMO's steeper trend line reflects RACMO's documented bias toward higher snow accumulation and a larger snow-covered area (Ryan et al, 2019, 2020). Consequently, grid cells classified as bare ice in our MODIS-derived bare ice mask occasionally correspond to snow-covered areas in RACMO, leading to a cluster of high-albedo values that artificially steepens RACMO's albedo-elevation trend. MAR does not exhibit this issue to the same extent due to its snowline being more consistent with MODIS observations (Ryan et al, 2019), thus reducing the presence of high-albedo, snow-covered MAR grid cells in Fig. S10. While this discrepancy emphasizes the importance of using consistent snowline delineations when attributing model differences in runoff to albedo processes at larger spatial scales such as the SW sector, this issue does not affect our comparison at the LG catchment scale (Fig. S10 panel c). Here, albedo and runoff patterns are consistent with those found at the smaller RB catchment scale, with RACMO albedo better aligned with MODIS, while MAR and MERRA-2 tend to overestimate MODIS albedo.

S4.2 Uncertainty in ice core density measurements

Ice-core densities were measured on ∼13 cm core sections after extraction and placement on the ice surface, using a digital balance and caliper-derived volumes. Core sections were weighed sequentially from the surface downward. During processing the exposed lower sections were exposed for a longer time, potentially allowing incidental

melt and drainage of intergranular water prior to weighing, which would lower their apparent density. Because these effects are unquantified, depth-dependent measurement error cannot be ruled out and may explain part of the model–data mismatch in ice density below ∼0.4 m, shown in Fig. 4b (Main).

S4.3 Uncertainty in Proglacial Discharge Comparisons

Relative to our direct supraglacial discharge measurements at RB catchment, proglacial discharge carries additional uncertainty due to uncertain upstream contributing area, potential englacial and subglacial meltwater contributions, and groundwater fluxes. Unlike supraglacial discharge, which is measured at the point of surface meltwater production, proglacial discharge integrates water from multiple sources, including surface runoff, englacial storage, subglacial drainage, and potentially groundwater inputs from the ice sheet bed. The upstream contributing area of proglacial rivers is not directly observable and depends on subglacial topography, which remains poorly constrained.

Catchment area uncertainty directly impacts model-data comparisons. For example, if the true LG catchment area were larger than assumed, all model runoff estimates would shift upward, potentially bringing LG into better agreement with results at other catchments. Conversely, if the catchment area were smaller, model estimates would shift downward, weakening agreement with observations at both LG and AK4. While we account for this uncertainty in Fig. 5c–d by varying assumed catchment area, it remains an inherent limitation in comparing proglacial discharge to surface energy balance models. Furthermore, englacial and subglacial water routing could contribute additional meltwater, though we assume these contributions are negligible relative to total runoff. While IceModel demonstrates a physically plausible improvement relative to SkinModel, its ability to predict actual proglacial discharge is still constrained by upstream area uncertainty and unknown englacial/subglacial contributions.

S4.4 Limitations of the Emulator Approach

Although factors other than albedo and refreezing likely contribute to model discrepancies at the LG catchment scale, a consistent bias in climate models toward overpredicting runoff suggests a missing retention mechanism. SkinModel was designed for the narrow purpose of emulating the surface energy balance method of computing meltwater runoff used by regional climate models (RCMs). While SkinModel emulates RCM runoff predictions well at RB catchment when forced with their respective albedo schemes, it is not expected to replicate them exactly across all times, scales, and locations.

In particular, differences in turbulent heat flux parameterizations across models, as well as numerical grid resolution, timestep, and other factors likely contribute to imperfect numerical emulation. This is reflected in our SkinModel simulations at LG catchment, where SkinModel forced with MAR does not fully replicate MAR runoff, contributing to ambiguity in this specific comparison. However, this discrepancy does not undermine the broader conclusion that IceModel better matches observations when compared to RCMs and to SkinModel. Rather, it underscores the importance of catchment delineation uncertainty in interpreting regional-scale runoff observations.

Finally, while performance attribution of IceModel relative to RCMs is complicated by unknown model structural differences, its improvement over SkinModel (Table S3) is directly attributable to the inclusion of subsurface radiative transfer, melting, and refreezing.

S5 Supplementary Runoff Comparison Figures

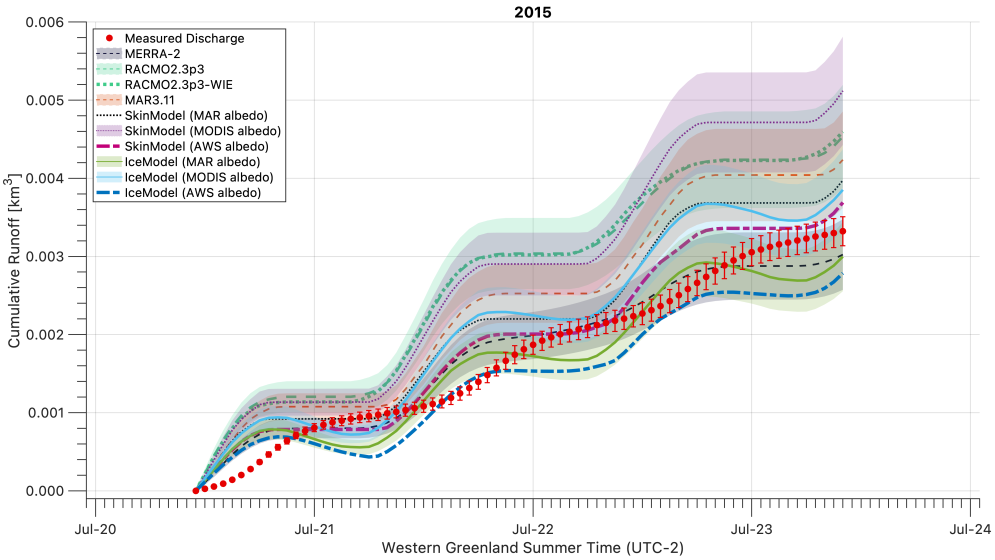

Fig. S15 Runoff comparison for RB catchment in year 2015. This comparison includes RACMO2.3p3 and the control version without internal energy (WIE) from subsurface radiative heating (Methods). AWS albedo for IceModel and SkinModel simulations is from KAN_M (Fig. S1).

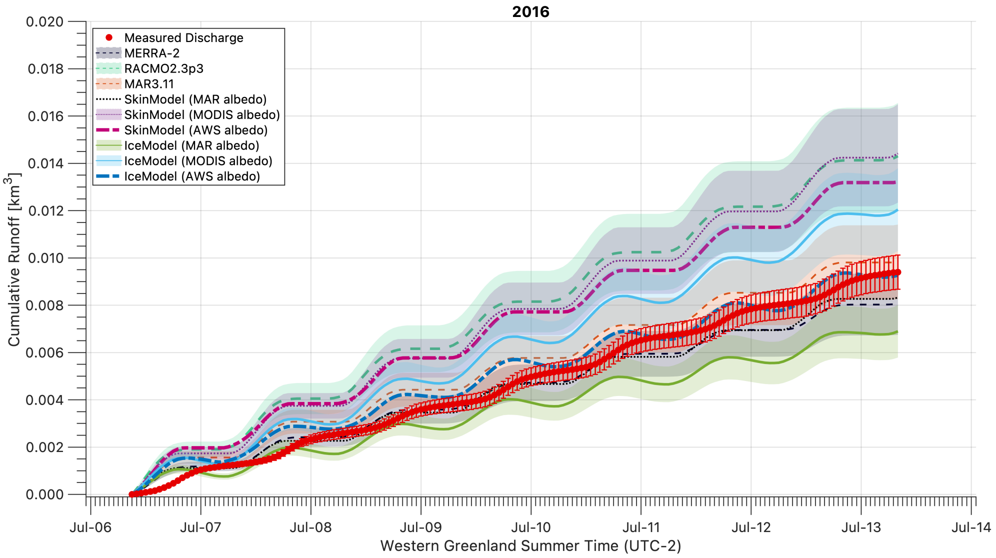

Fig. S16 Runoff comparison for RB catchment in year 2016. AWS albedo for IceModel and SkinModel simulations is from KAN_M (Fig. S1).

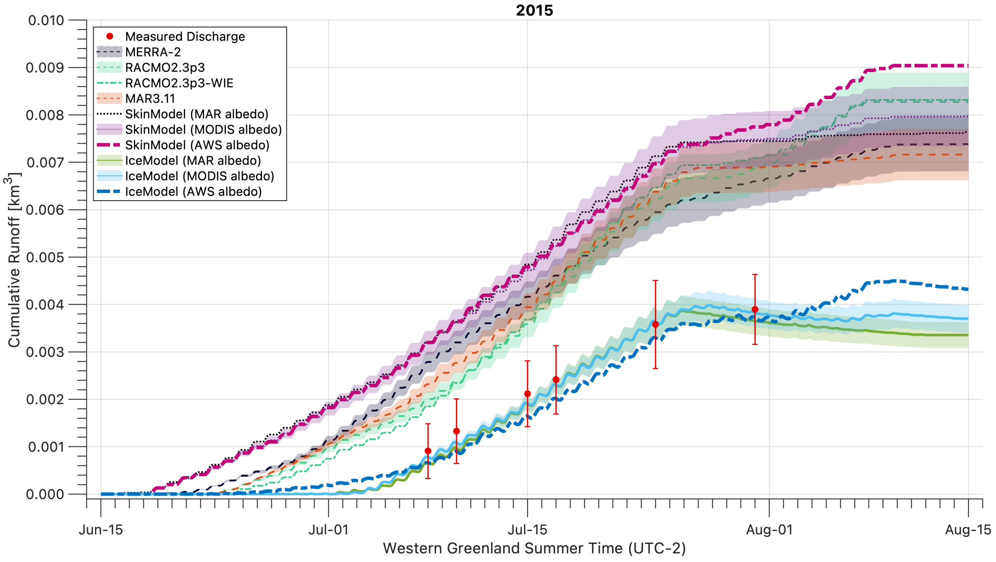

Fig. S17 Runoff comparison for SLV1 catchment in year 2015. This comparison includes RACMO2.3p3 and the control version without internal energy (WIE) from subsurface radiative heating (see Methods). AWS albedo for IceModel and SkinModel simulations is from KAN_M (Fig. S1).

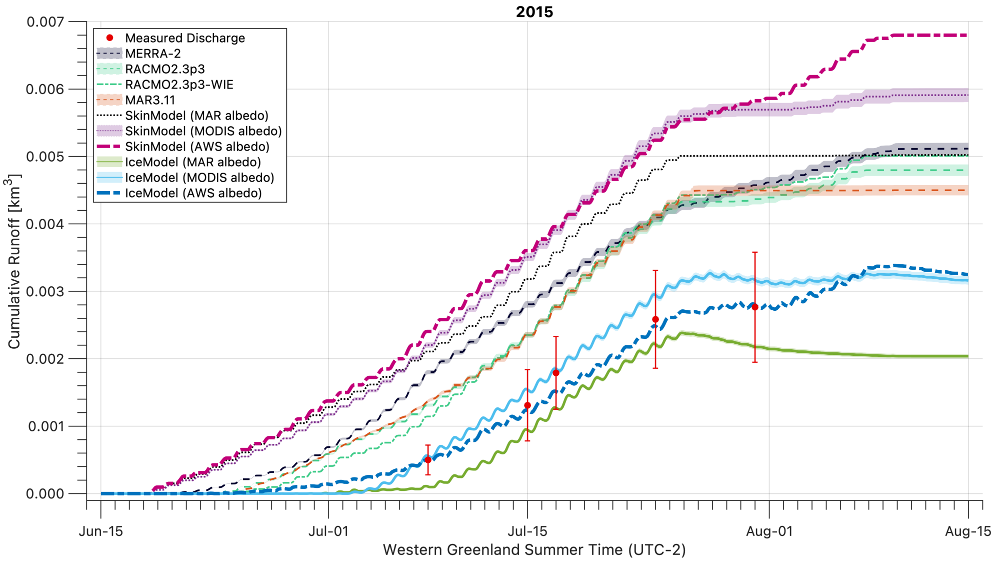

Fig. S18 Runoff comparison for SLV2 catchment in year 2015. This comparison includes RACMO2.3p3 and the control version without internal energy (WIE) from subsurface radiative heating (see Methods). AWS albedo for IceModel and SkinModel simulations is from KAN_M (Fig. S1).

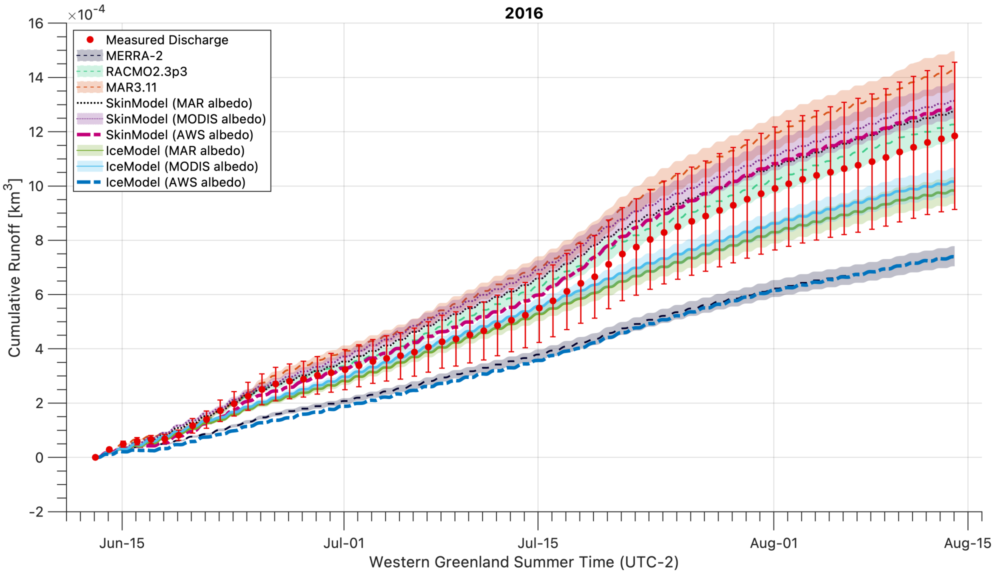

Fig. S19 Runoff comparison for 660 catchment in year 2016. AWS albedo for IceModel and SkinModel simulations is from KAN_L (Fig. S1).

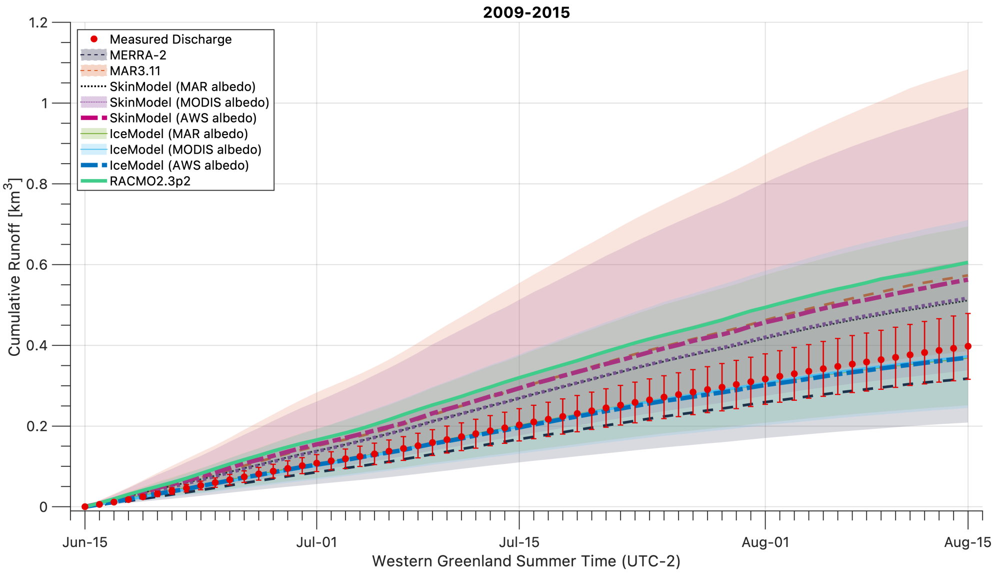

Fig. S20 Runoff comparison for AK4 catchment over years 2009–2015. Here, runoff values are summed over each year in the period to compute the total runoff discharged over the period. RACMO2.3p3 is omitted due to data availability for years 2009–2011 (see Fig. S24–S27 for RACMO2.3p3 comparison in years 2012-2015). Runoff comparisons for each year in 2009–2015 are shown in Fig. S21–S27. AWS albedo for IceModel and SkinModel simulations is from KAN_L (Fig. S1).

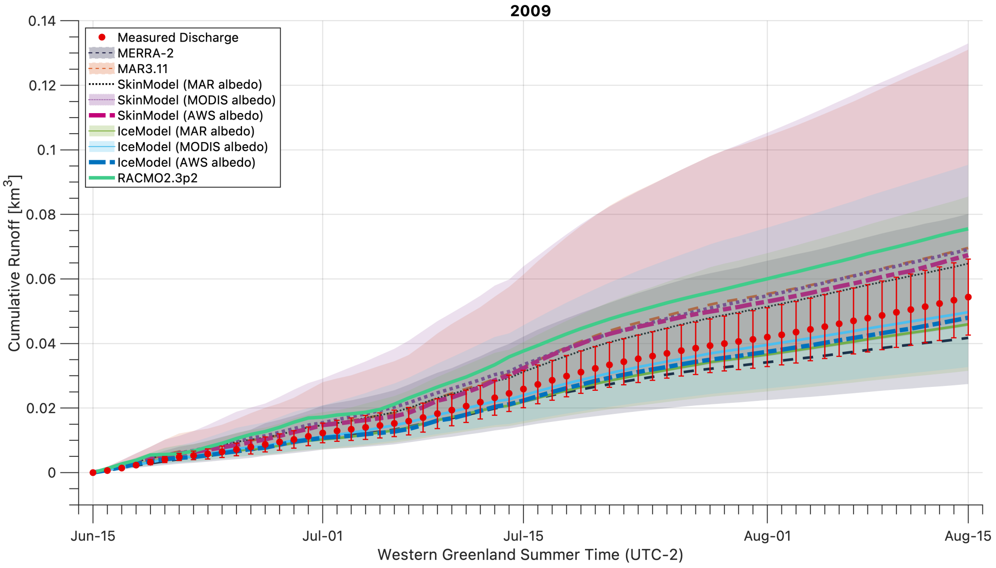

Fig. S21 Runoff comparison for AK4 catchment in year 2009. AWS albedo for IceModel and SkinModel simulations is from KAN_L (Fig. S1).

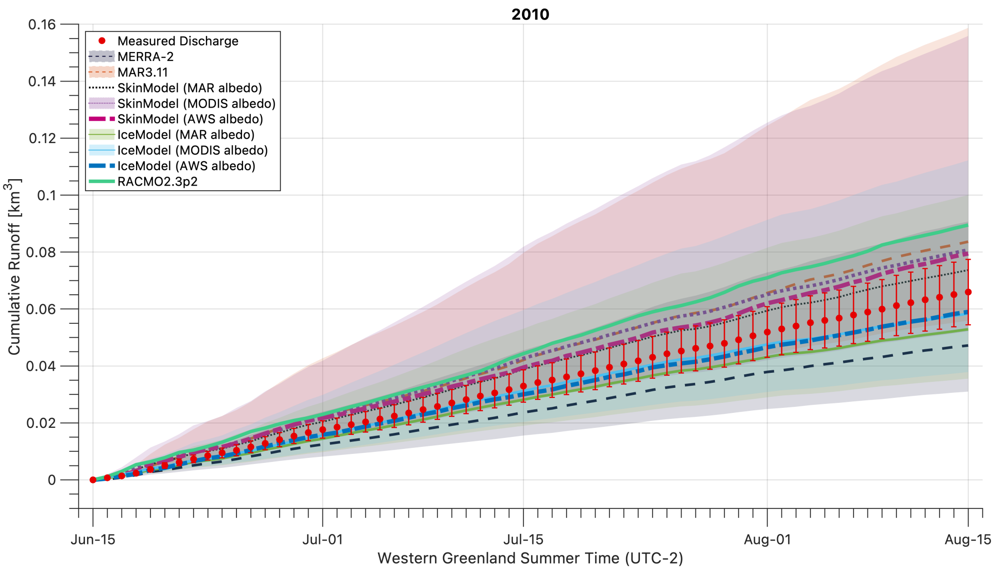

Fig. S22 Runoff comparison for AK4 catchment in year 2010. AWS albedo for IceModel and SkinModel simulations is from KAN_L (Fig. S1).

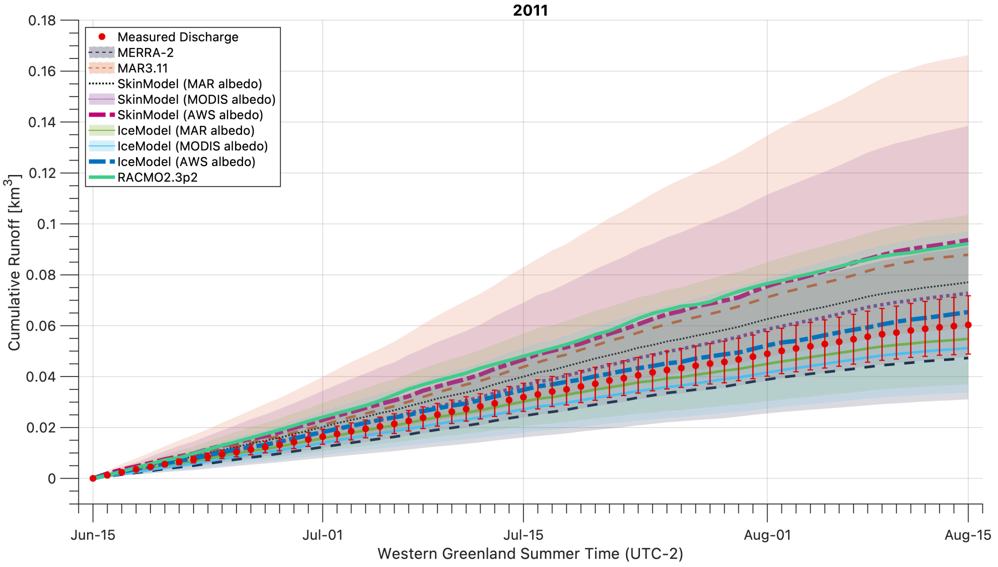

Fig. S23 Runoff comparison for AK4 catchment in year 2011. AWS albedo for IceModel and SkinModel simulations is from KAN_L (Fig. S1).

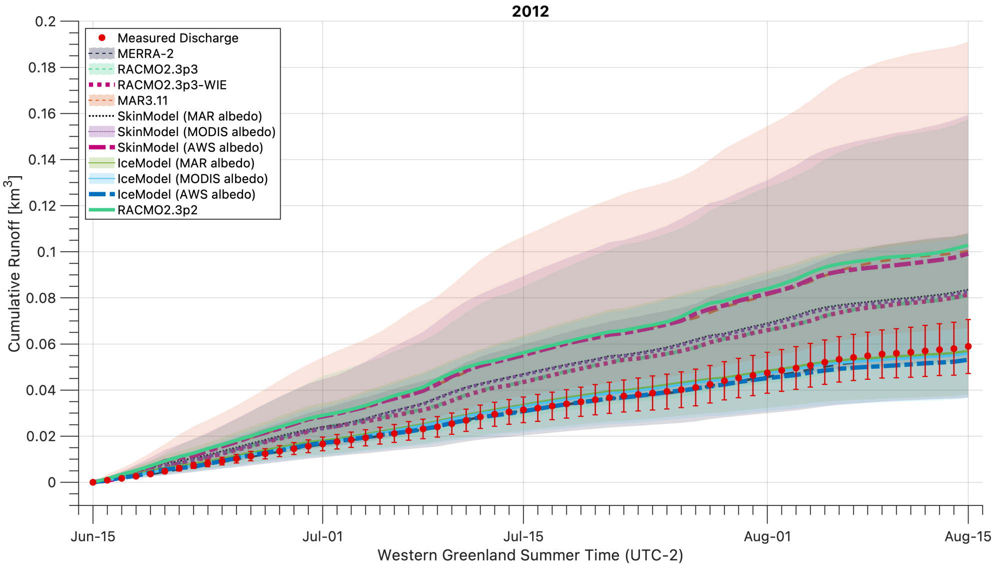

Fig. S24 Runoff comparison for AK4 catchment in year 2012. This comparison includes RACMO2.3p3 and the control version without internal energy (WIE) from subsurface radiative heating (see Methods). AWS albedo for IceModel and SkinModel simulations is from KAN_L (Fig. S1).

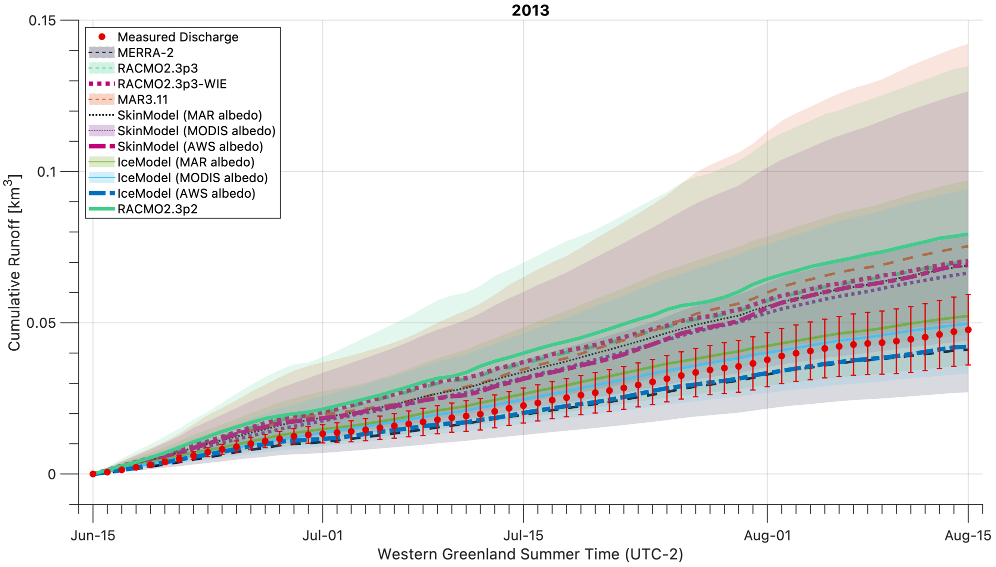

Fig. S25 Runoff comparison for AK4 catchment in year 2013. This comparison includes RACMO2.3p3 and the control version without internal energy (WIE) from subsurface radiative heating (see Methods). AWS albedo for IceModel and SkinModel simulations is from KAN_L (Fig. S1).

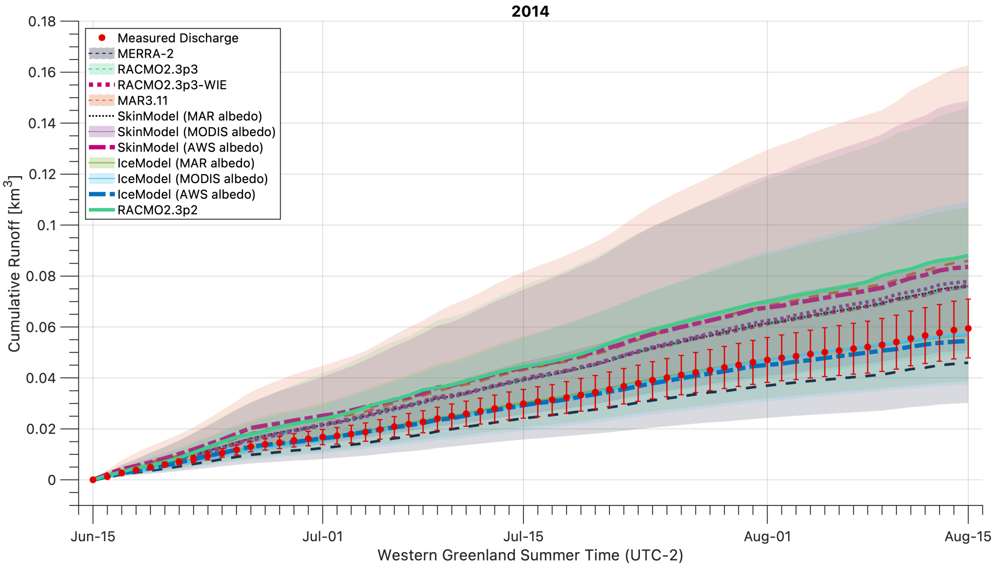

Fig. S26 Runoff comparison for AK4 catchment in year 2014. This comparison includes RACMO2.3p3 and the control version without internal energy (WIE) from subsurface radiative heating (see Methods). AWS albedo for IceModel and SkinModel simulations is from KAN_L (Fig. S1).

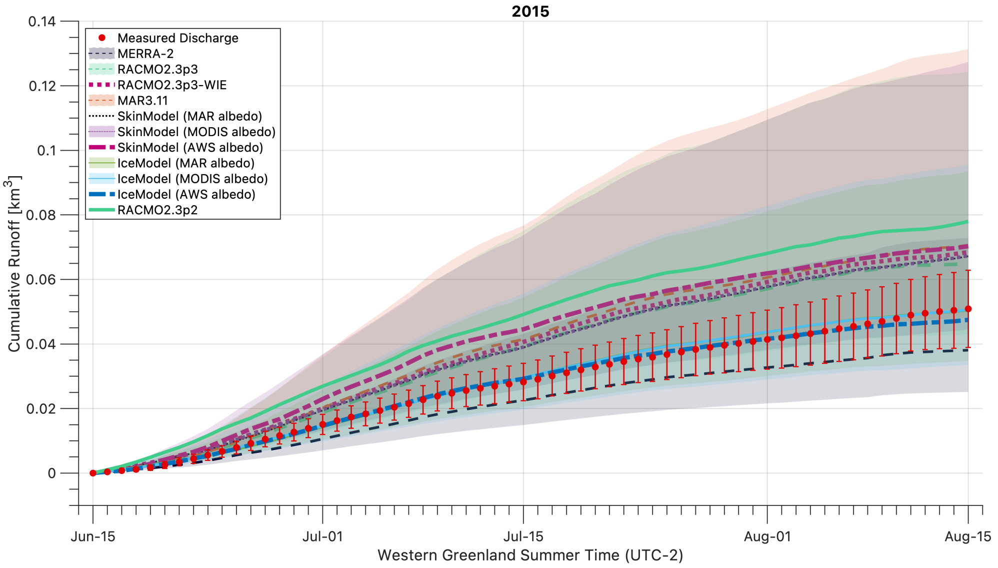

Fig. S27 Runoff comparison for AK4 catchment in year 2015. This comparison includes RACMO2.3p3 and the control version without internal energy (WIE) from subsurface radiative heating (see Methods). AWS albedo for IceModel and SkinModel simulations is from KAN_L (Fig. S1).

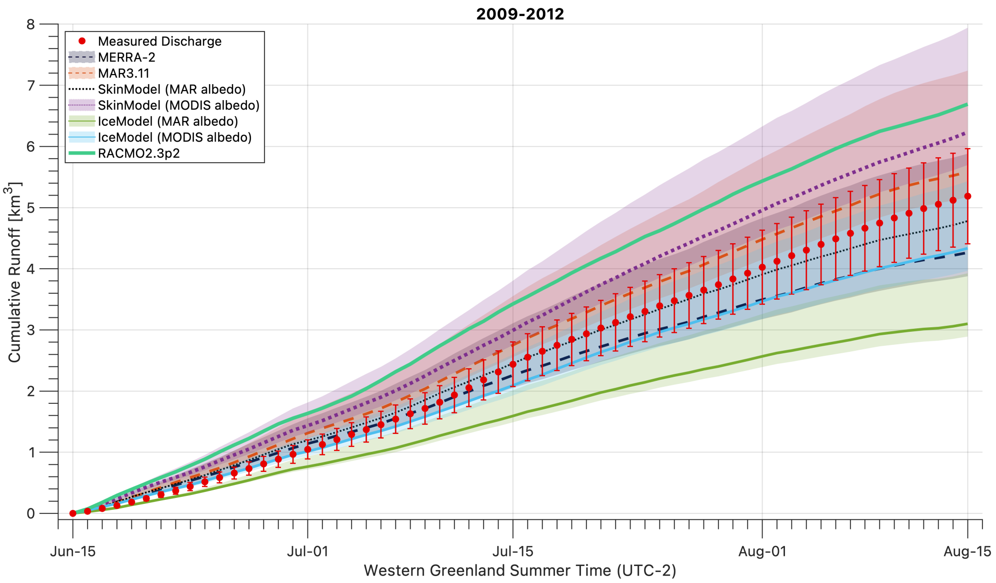

Fig. S28 Runoff comparison for LG catchment over years 2009–2012. Here, runoff values are summed over each year in the period to compute the total runoff discharged over the period. The LG runoff comparison exclusively includes RACMO2.3p2, while RACMO2.3p3 is omitted due to data availability for years 2009–2011 (see Fig. S32 for RACMO2.3p3 comparison in year 2012). Runoff comparisons for each year in 2009–2012 are shown in Fig. S29–S32.

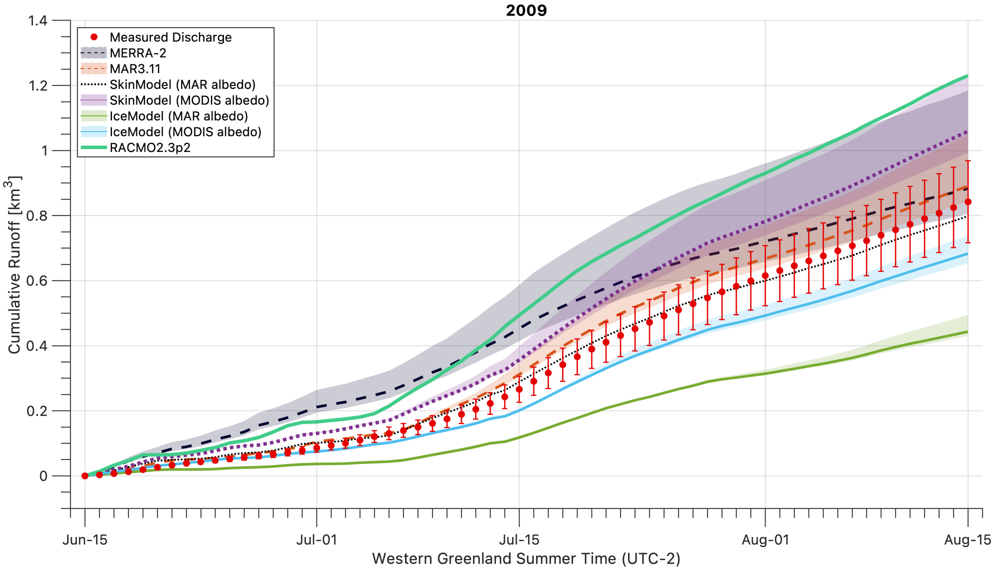

Fig. S29 Runoff comparison for LG catchment in year 2009. This comparison includes RACMO2.3p2, while RACMO2.3p3 is omitted due to data availability for years 2009–2011 (see Fig. S32 for RACMO2.3p3 comparison in year 2012).

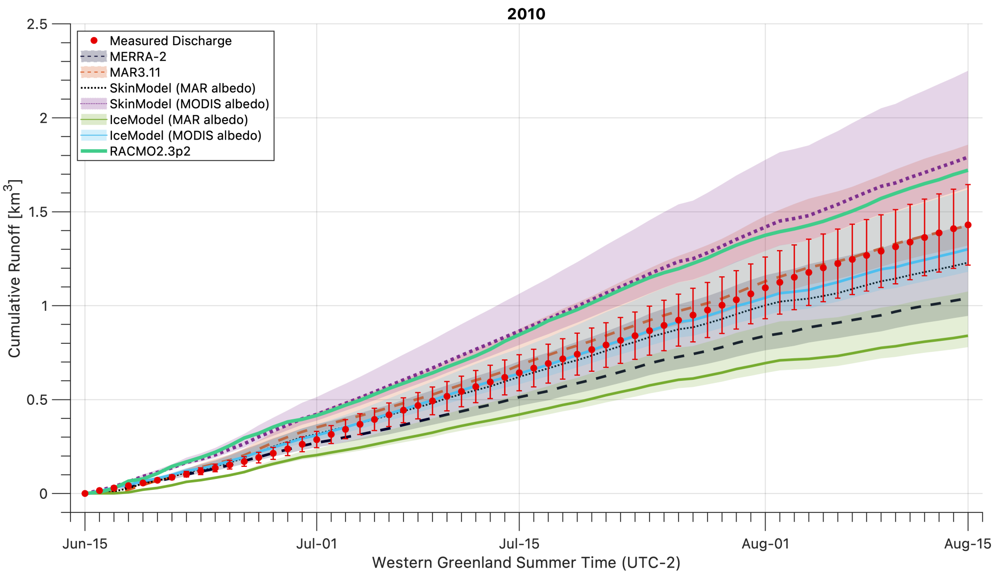

Fig. S30 Runoff comparison for LG catchment in year 2010. This comparison includes RACMO2.3p2, while RACMO2.3p3 is omitted due to data availability for years 2009–2011 (see Fig. S32 for RACMO2.3p3 comparison in year 2012).

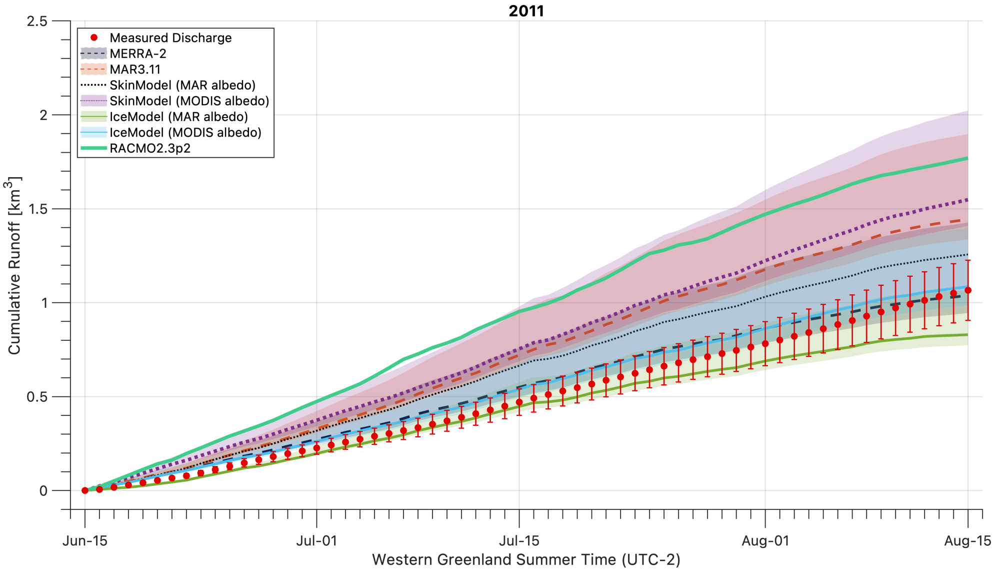

Fig. S31 Runoff comparison for LG catchment in year 2011. This comparison includes RACMO2.3p2, while RACMO2.3p3 is omitted due to data availability for years 2009–2011 (see Fig. S32 for RACMO2.3p3 comparison in year 2012).

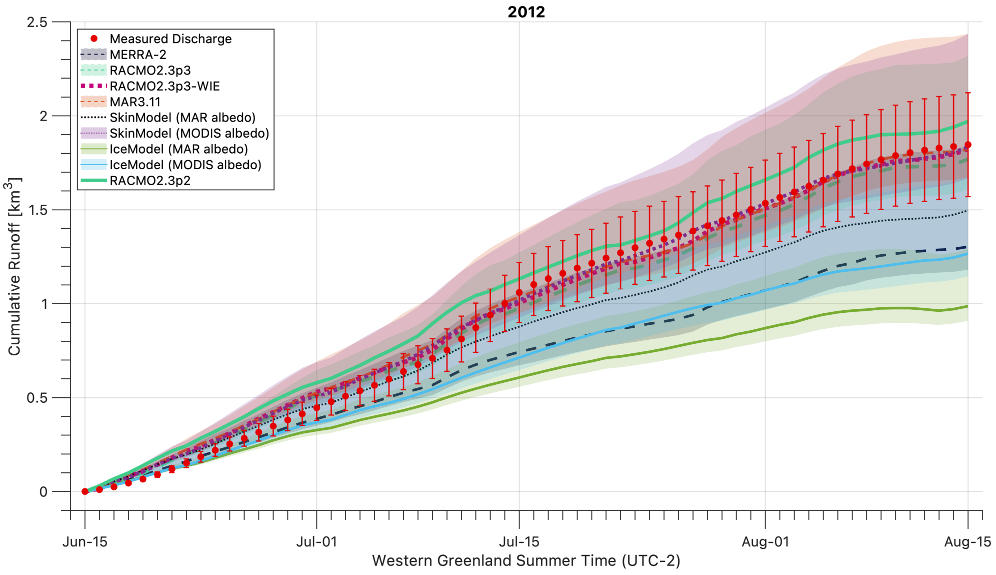

Fig. S32 Runoff comparison for LG catchment in year 2012. This comparison includes RACMO2.3p3 and the control version without internal energy (WIE) from subsurface radiative heating (see Methods).

S6 Supplementary Remapping Weight Figures

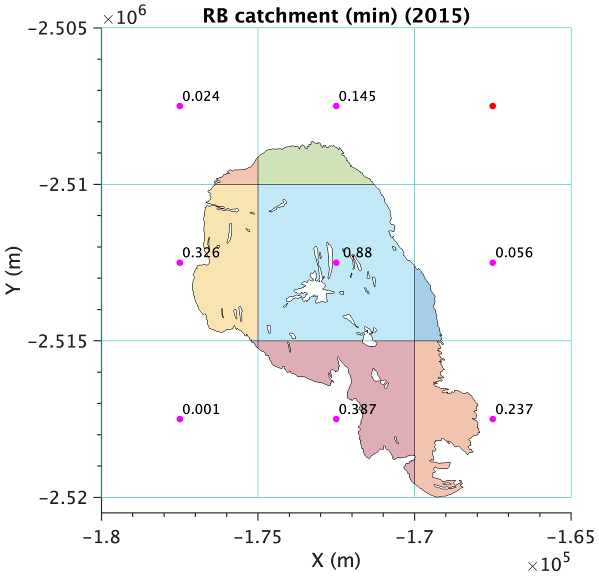

Fig. S33 Remapping weights for RB catchment minimum estimated boundary in year 2015.

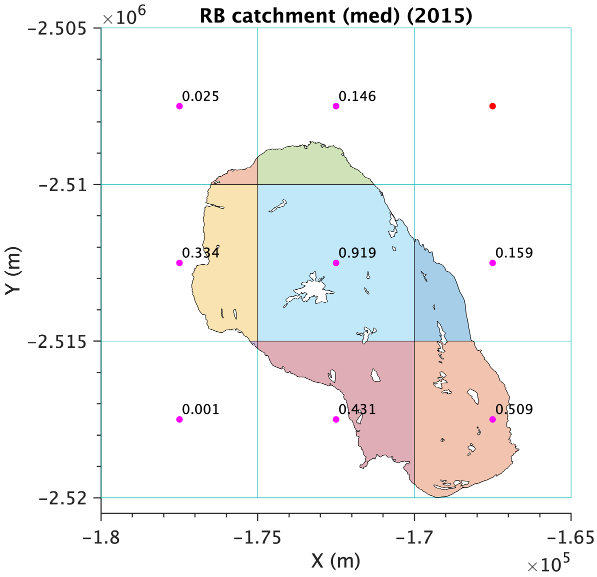

Fig. S34 Remapping weights for RB catchment medium estimated boundary in year 2015.

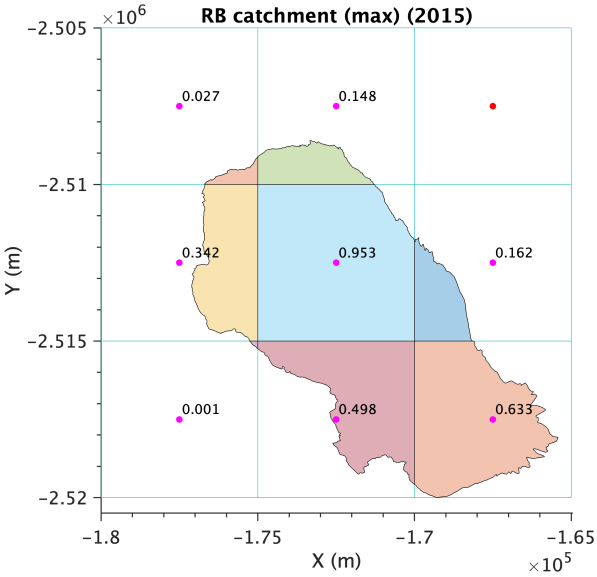

Fig. S35 Remapping weights for RB catchment maximum estimated boundary in year 2015.

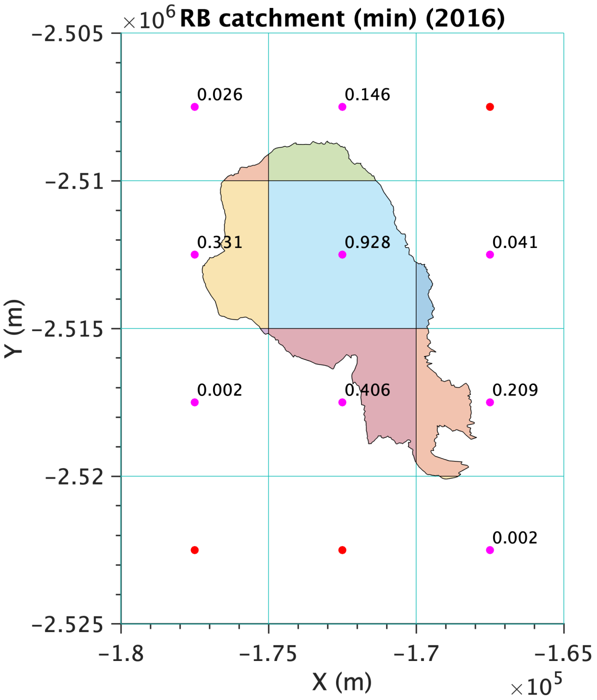

Fig. S36 Remapping weights for RB catchment minimum estimated boundary in year 2016.

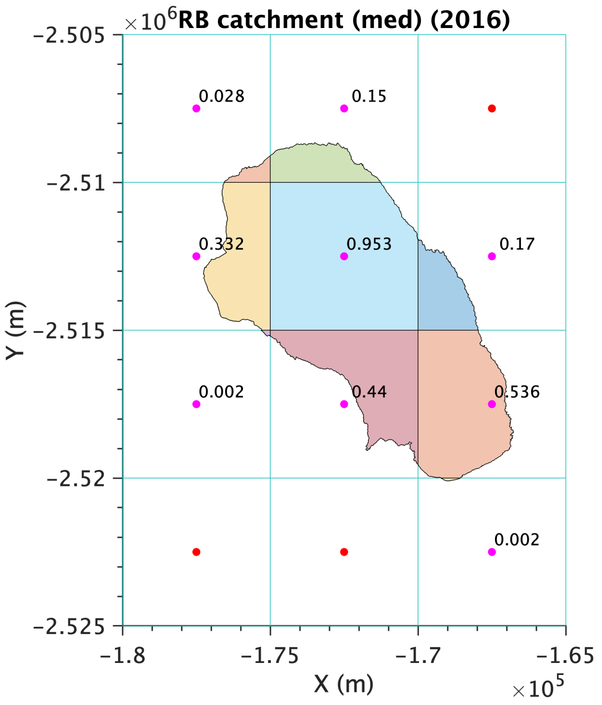

Fig. S37 Remapping weights for RB catchment medium estimated boundary in year 2016.

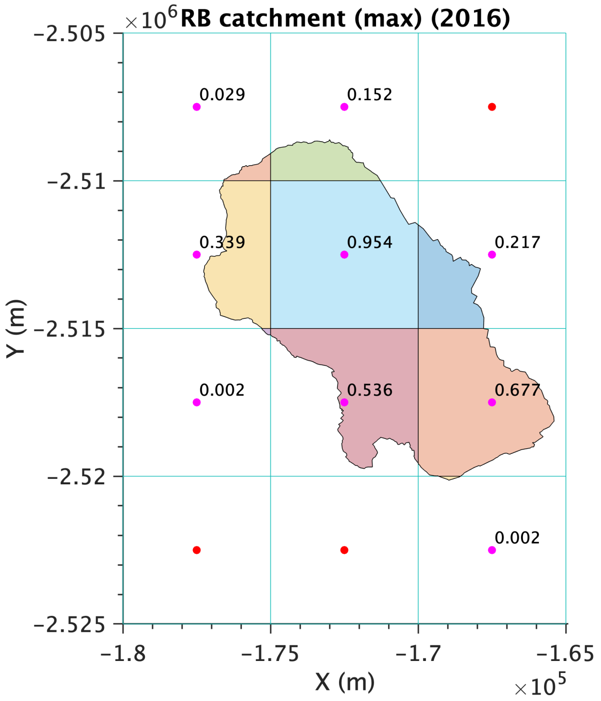

Fig. S38 Remapping weights for RB catchment maximum estimated boundary in year 2016.

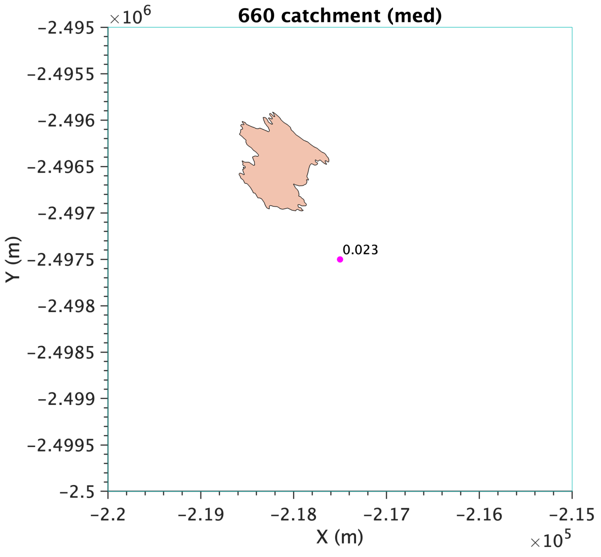

Fig. S39 Remapping weights for 660 catchment medium estimated boundary in year 2016.

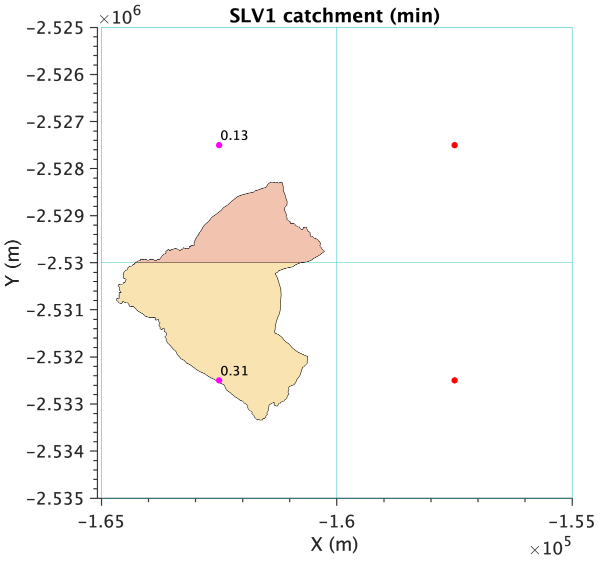

Fig. S40 Remapping weights for SLV1 catchment minimum estimated boundary in year 2015.

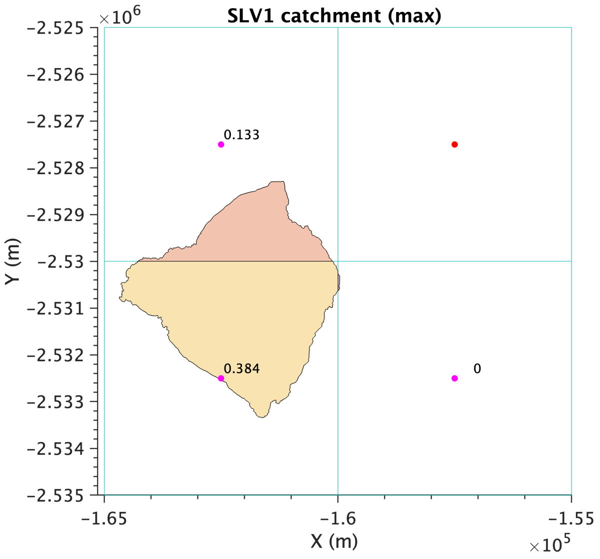

Fig. S41 Remapping weights for SLV1 catchment maximum estimated boundary in year 2015.

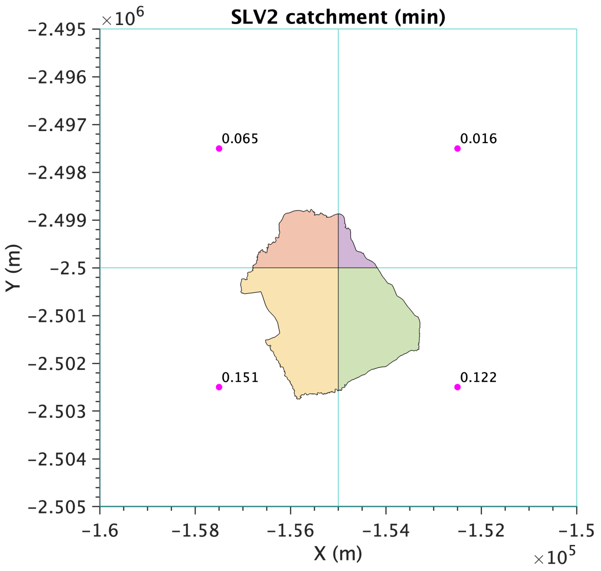

Fig. S42 Remapping weights for SLV2 catchment minimum estimated boundary in year 2015.

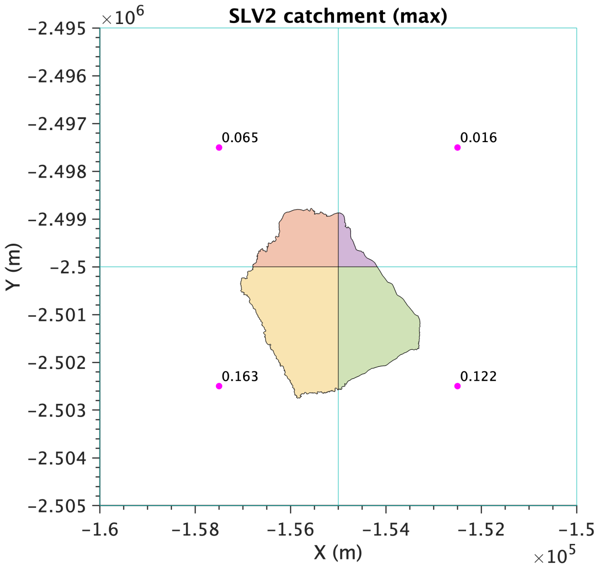

Fig. S43 Remapping weights for SLV2 catchment maximum estimated boundary in year 2015.

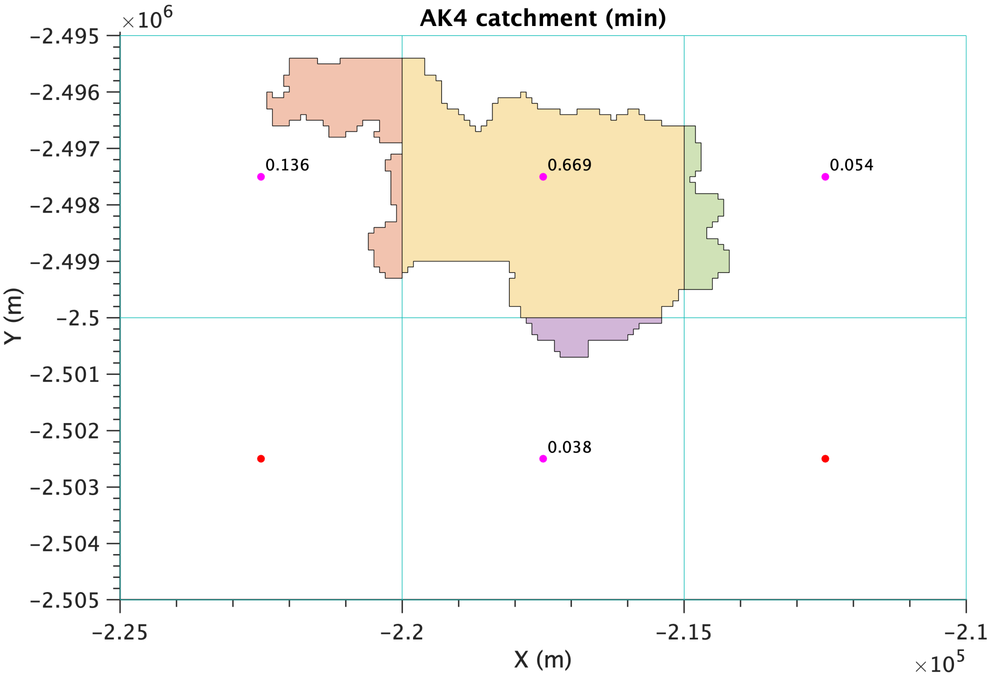

Fig. S44 Remapping weights for AK4 catchment minimum estimated boundary for years 2009–2015.

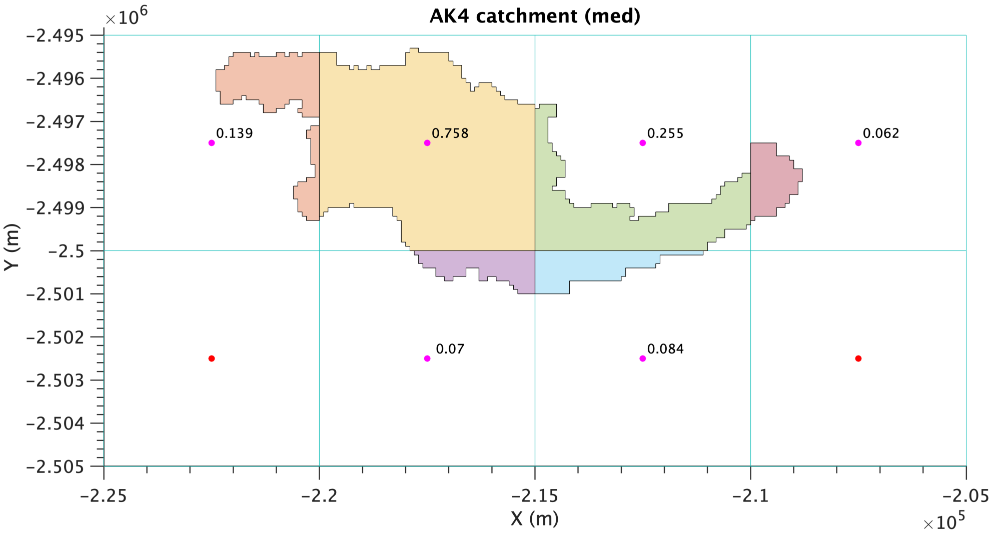

Fig. S45 Remapping weights for AK4 catchment medium estimated boundary for years 2009–2015.

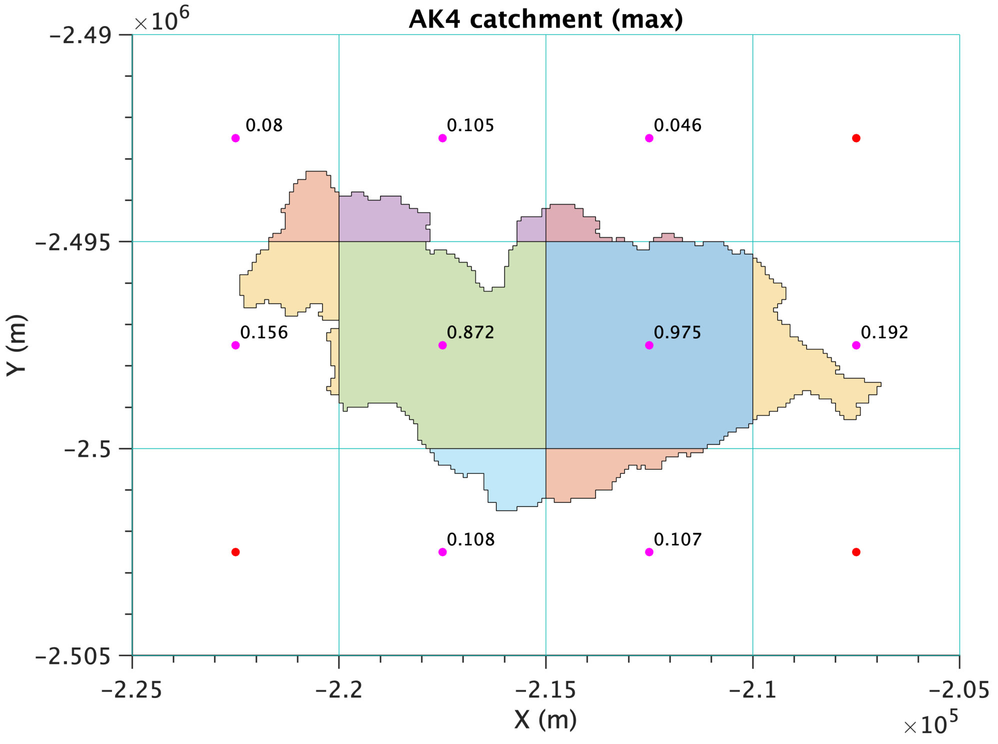

Fig. S46 Remapping weights for AK4 catchment maximum estimated boundary for years 2009–2015.

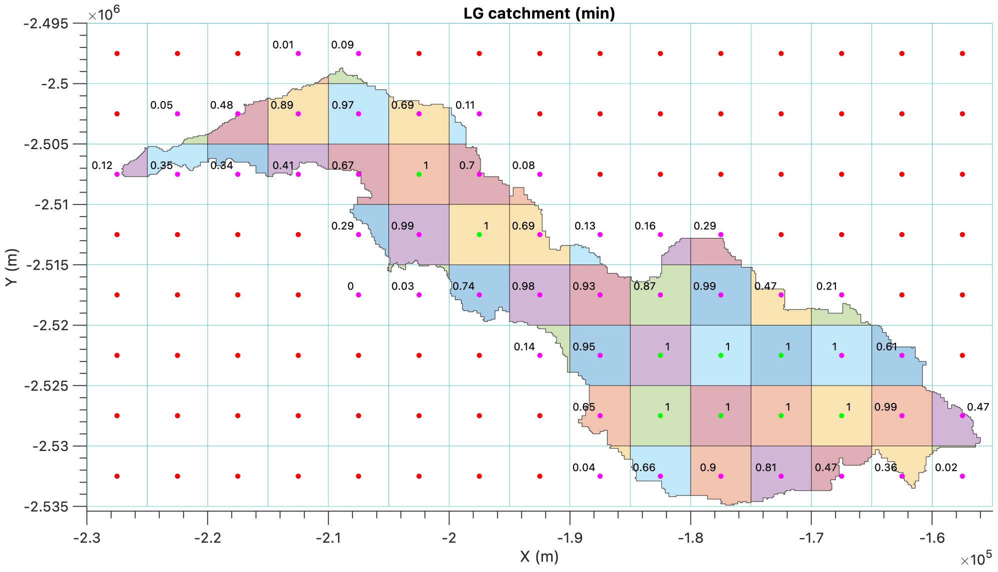

Fig. S47 Remapping weights for LG catchment minimum estimated boundary.

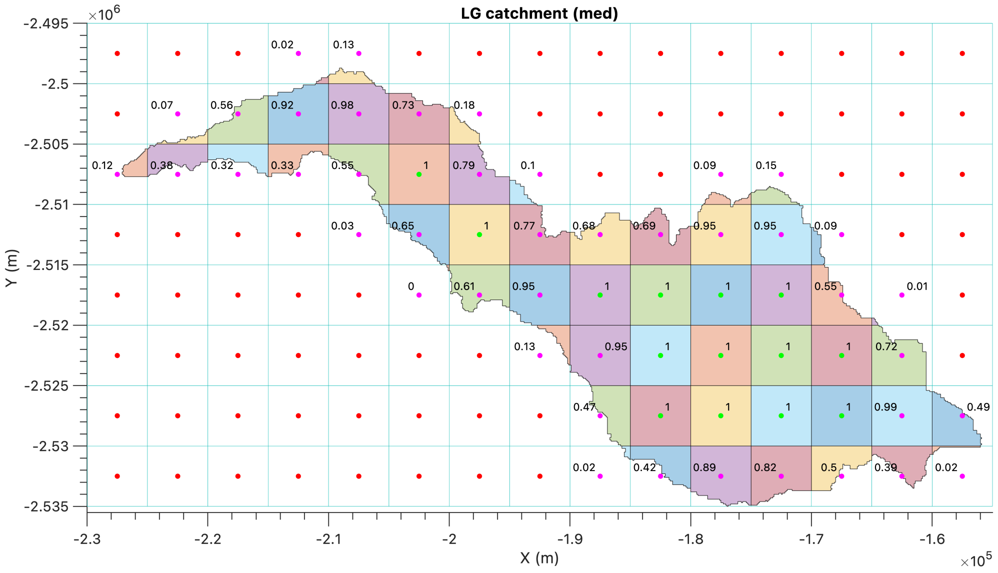

Fig. S48 Remapping weights for LG catchment medium estimated boundary for years 2009–2012.

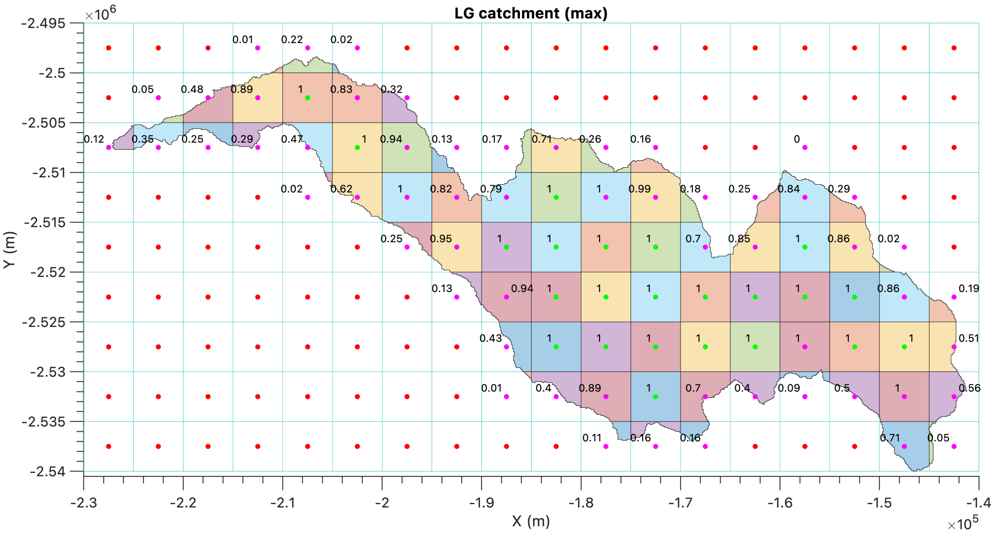

Fig. S49 Remapping weights for LG catchment maximum estimated boundary for years 2009–2012.

References

Anderson EA (1976) A point energy and mass balance model of a snow cover. Technical Report 19, Office of Hydrology, National Weather Service

Bohren CF, Huffman DR (2007) Absorption and Scattering of Light by Small Particles. John Wiley & Sons, Ltd

Box JE, van As D, Steffen K (2017) Greenland, Canadian and Icelandic land-ice albedo grids (2000–2016). GEUS Bulletin 38:53–56. https://doi.org/10.34194/geusb.v38.4414

Brandt RE, Warren SG (1993) Solar-heating rates and temperature profiles in Antarctic snow and ice. Journal of Glaciology 39(131):99–110. https://doi.org/10.3189/S0022143000015756

Brodzik MJ, Billingsley B, Haran T, et al (2012) EASE-Grid 2.0: Incremental but Significant Improvements for Earth-Gridded Data Sets. ISPRS International Journal of Geo-Information 1(1):32–45. https://doi.org/10.3390/ijgi1010032

Buck AL (1981) New Equations for Computing Vapor Pressure and Enhancement Factor. Journal of Applied Meteorology 20(12):1527–1532. https://doi.org/10.1175/1520-0450(1981)020<1527:NEFCVP>2.0.CO;2

Calonne N, Milliancourt L, Burr A, et al (2019) Thermal Conductivity of Snow, Firn, and Porous Ice From 3-D Image-Based Computations. Geophysical Research Letters 46(22):13079–13089. https://doi.org/10.1029/2019GL085228

Clark MP, Zolfaghari R, Green KR, et al (2021) The Numerical Implementation of Land Models: Problem Formulation and Laugh Tests. Journal of Hydrometeorology 22(6):1627–1648. https://doi.org/10.1175/JHM-D-20-0175.1

Cooper MG, Smith LC, Rennermalm ÅK, et al (2018) Meltwater storage in low-density near-surface bare ice in the Greenland ice sheet ablation zone. The Cryosphere 12(3):955–970. https://doi.org/10.5194/tc-12-955-2018

Cooper MG, Smith LC, Rennermalm ÅK, et al (2021) Spectral attenuation coefficients from measurements of light transmission in bare ice on the Greenland Ice Sheet. The Cryosphere 15(4):1931–1953. https://doi.org/10.5194/tc-15-1931-2021

Fausto RS, van As D (2019) Programme for monitoring of the Greenland ice sheet (PROMICE): Automatic weather station data. https://doi.org/10.22008/PROMICE/DATA/AWS

Fettweis X, Box JE, Agosta C, et al (2017) Reconstructions of the 1900–2015 Greenland ice sheet surface mass balance using the regional climate MAR model. The Cryosphere 11(2):1015–1033. https://doi.org/10.5194/tc-11-1015-2017

Fettweis X, Hofer S, Krebs-Kanzow U, et al (2020) GrSMBMIP: Intercomparison of the modelled 1980–2012 surface mass balance over the Greenland Ice Sheet. The Cryosphere 14(11):3935–3958. https://doi.org/10.5194/tc-14-3935-2020

Gelaro R, McCarty W, Suárez MJ, et al (2017) The Modern-Era Retrospective Analysis for Research and Applications, Version 2 (MERRA-2). Journal of Climate 30(14):5419–5454. https://doi.org/10.1175/JCLI-D-16-0758.1

Hills BH, Harper JT, Meierbachtol TW, et al (2018) Processes influencing heat transfer in the near-surface ice of Greenland's ablation zone. The Cryosphere 12(10):3215–3227. https://doi.org/10.5194/tc-12-3215-2018

Jordan R (1991) A One-Dimensional Temperature Model For a Snowpack. Special Report 91-16, Cold Regions Research and Engineering Laboratory, Hanover, NH

Liston GE, Bruland O, Elvehøy H, et al (1999) Below-surface ice melt on the coastal Antarctic ice sheet. Journal of Glaciology 45(150):273–285. https://doi.org/10.3189/002214399793377130

Mankoff KD, Noël B, Fettweis X, et al (2020) Greenland liquid water discharge from 1958 through 2019. Earth System Science Data 12(4):2811–2841. https://doi.org/10.5194/essd-12-2811-2020

Mätzler C (2002) MATLAB Functions for Mie Scattering and Absorption, Version 2. Research Report 2002-11, Institut für Angewandte Physik, Bern, Switzerland. https://boris.unibe.ch/146550/, https://doi.org/10.7892/BORIS.146550

Muthyala R, Rennermalm ÅK, Leidman SZ, et al (2022) Supraglacial streamflow and meteorological drivers from southwest Greenland. The Cryosphere 16(6):2245–2263. https://doi.org/10.5194/tc-16-2245-2022

Patankar SV (1980) Numerical Heat Transfer and Fluid Flow. Series in Computational Methods in Mechanics and Thermal Sciences, Hemisphere Publishing Corporation

Rennermalm ÅK, Smith LC, Hammann AC, et al (2017) River discharge at station AK-004-001, 2008 - 2016, version 3.0. https://doi.org/10.1594/PANGAEA.876357

Ryan JC, Smith LC, van As D, et al (2019) Greenland Ice Sheet surface melt amplified by snowline migration and bare ice exposure. Science Advances 5(3):eaav3738. https://doi.org/10.1126/sciadv.aav3738

Ryan JC, Smith LC, Wu M, et al (2020) Evaluation of CloudSat's Cloud-Profiling Radar for Mapping Snowfall Rates Across the Greenland Ice Sheet. Journal of Geophysical Research: Atmospheres 125(4):e2019JD031411. https://doi.org/10.1029/2019JD031411

Schlatter TW (1972) The Local Surface Energy Balance and Subsurface Temperature Regime in Antarctica. Journal of Applied Meteorology 11(7):1048–1062. https://doi.org/10.1175/1520-0450(1972)011<1048:TLSEBA>2.0.CO;2

Shepherd A, Ivins E, Rignot E, et al (2020) Mass balance of the Greenland Ice Sheet from 1992 to 2018. Nature 579(7798):233–239. https://doi.org/10.1038/s41586-019-1855-2

Smith LC, Yang K, Pitcher LH, et al (2017) Direct measurements of meltwater runoff on the Greenland ice sheet surface. Proceedings of the National Academy of Sciences 114(50):E10622–E10631. https://doi.org/10.1073/pnas.1707743114

Smith LC, Andrews LC, Pitcher LH, et al (2021) Supraglacial River Forcing of Subglacial Water Storage and Diurnal Ice Sheet Motion. Geophysical Research Letters 48(7):e2020GL091418. https://doi.org/10.1029/2020GL091418

Swaminathan C, Voller V (1993) ON THE ENTHALPY METHOD. International Journal of Numerical Methods for Heat & Fluid Flow 3(3):233–244. https://doi.org/10.1108/eb017528

Tedstone AJ, Bartholomew I, Chandler D, et al (2017) Proglacial discharge measurements, Leverett Glacier, south-west Greenland (2009-2012). https://doi.org/10.5285/17c400f1-ed6d-4d5a-a51f-aad9ee61ce3d

van As D (2011) Warming, glacier melt and surface energy budget from weather station observations in the Melville Bay region of northwest Greenland. Journal of Glaciology 57(202):208–220. https://doi.org/10.3189/002214311796405898

van Dalum CT, van de Berg WJ, van den Broeke MR (2021) Impact of updated radiative transfer scheme in snow and ice in RACMO2.3p3 on the surface mass and energy budget of the Greenland ice sheet. The Cryosphere 15(4):1823–1844.

https://doi.org/10.5194/tc-15-1823-2021

Warren SG, Brandt RE (2008) Optical constants of ice from the ultraviolet to the microwave: A revised compilation. Journal of Geophysical Research: Atmospheres 113(D14):D14220. https://doi.org/10.1029/2007JD009744

Warren SG, Brandt RE, Grenfell TC (2006) Visible and near-ultraviolet absorption spectrum of ice from transmission of solar radiation into snow. Applied Optics 45(21):5320–5334. https://doi.org/10.1364/AO.45.005320

Yang K, Smith LC, Fettweis X, et al (2019) Surface meltwater runoff on the Greenland ice sheet estimated from remotely sensed supraglacial lake infilling rate. Remote Sensing of Environment 234:111459. https://doi.org/10.1016/j.rse.2019.111459
